# Predicting the impact of Lynch syndrome-causing missense mutations from structural calculations

**DOI:** 10.1371/journal.pgen.1006739

**Published:** 2017-04-19

**Authors:** Sofie V. Nielsen, Amelie Stein, Alexander B. Dinitzen, Elena Papaleo, Michael H. Tatham, Esben G. Poulsen, Maher M. Kassem, Lene J. Rasmussen, Kresten Lindorff-Larsen, Rasmus Hartmann-Petersen

**Affiliations:** 1 The Linderstrøm-Lang Centre for Protein Science, Department of Biology, University of Copenhagen, Ole Maaløes Vej 5, Copenhagen, Denmark; 2 Computational Biology Laboratory, Unit of Statistics, Bioinformatics and Registry, Danish Cancer Society Research Center, Strandboulevarden 49, Copenhagen, Denmark; 3 Centre for Gene Regulation and Expression, Sir James Black Centre, College of Life Sciences, University of Dundee, Dundee, United Kingdom; 4 Center for Healthy Aging, Department of Cellular and Molecular Medicine, University of Copenhagen, Blegdamsvej 3B, Copenhagen, Denmark; St. Jude Children's Research Hospital, UNITED STATES

## Abstract

Accurate methods to assess the pathogenicity of mutations are needed to fully leverage the possibilities of genome sequencing in diagnosis. Current data-driven and bioinformatics approaches are, however, limited by the large number of new variations found in each newly sequenced genome, and often do not provide direct mechanistic insight. Here we demonstrate, for the first time, that saturation mutagenesis, biophysical modeling and co-variation analysis, performed *in silico*, can predict the abundance, metabolic stability, and function of proteins inside living cells. As a model system, we selected the human mismatch repair protein, MSH2, where missense variants are known to cause the hereditary cancer predisposition disease, known as Lynch syndrome. We show that the majority of disease-causing MSH2 mutations give rise to folding defects and proteasome-dependent degradation rather than inherent loss of function, and accordingly our *in silico* modeling data accurately identifies disease-causing mutations and outperforms the traditionally used genetic disease predictors. Thus, in conclusion, *in silico* biophysical modeling should be considered for making genotype-phenotype predictions and for diagnosis of Lynch syndrome, and perhaps other hereditary diseases.

## Author summary

The protein quality control system targets misfolded proteins for degradation. So far it has not been possible from sequence or structural data to predict the biological stability of a misfolded protein, or the effect of mutations on intracellular protein levels. Here we demonstrate that *in silico* saturation mutagenesis and biophysical calculations of the structural stability of the human mismatch repair protein MSH2 correlate with cellular protein levels, turnover and function. Of 24 different MSH2 variants, some of which are linked to Lynch syndrome, a destabilization of as little as 3 kcal/mol is sufficient to cause rapid degradation via the ubiquitin-proteasome pathway. Thus, biophysical modeling can, to a large extent, predict the metabolic stability of proteins. We also show that the same biophysical calculations can be used to distinguish with high accuracy neutral sequence variation from pathogenic variants, and that the calculations outperform several traditionally used disease predictors. We therefore suggest the method to be of potential value for patient stratification in Lynch syndrome, and perhaps other hereditary diseases.

## Introduction

Due to mutations, stress, or failures during synthesis, cells produce proteins that misfold. Accumulation of misfolded proteins represents a considerable threat to cells, which have therefore evolved efficient protein quality control (PQC) mechanisms [1–3]. These rely on molecular chaperones that either refold the misfolded proteins or target them for degradation via the ubiquitin-proteasome system (UPS).

Early studies showed that certain missense protein variants are more rapidly degraded than wild type proteins [4]. Since then a number of proteins involved in targeting the misfolded proteins for degradation have been identified, particularly in yeast cells, where mutants in UPS components were identified as extragenic suppressors of point mutants in essential genes [5,6]. These observations suggest that PQC is highly diligent and important, but the issue of what determines whether a mutant protein is degraded or not remains unanswered.

To further our understanding on the intricate relationship between protein stability, degradation and biological function, we performed *in silico* and cellular studies of the mismatch repair protein MSH2, which has previously been shown to be a target of a PQC pathway in yeast cells [7]. Point mutations in *MSH2* are linked to hereditary nonpolyposis colorectal cancer (HNPCC) or Lynch syndrome, an inherited disorder that increases the risk of many types of cancer, in particular colon cancer [8]. Identification of pathogenic MSH2 mutations would be of direct clinical relevance, because an early diagnosis can strongly increase survival [9], but many mutations are of unknown pathogenic significance. We found that the predicted structural stability of MSH2 correlates with the cellular protein stability, but even slight structural perturbations may result in MSH2 degradation. Treating cells with the proteasome inhibitor bortezomib or stabilizing MSH2 mutants by lowering the temperature strongly reduced MSH2 degradation, showing that the proteasomal degradation of MSH2 variants is a direct consequence of a structural destabilization. Thus, in conclusion our data show for the first time that biophysical modelling can predict the stability of proteins in cells and suggest that biophysical modelling can provide both mechanistic insight and a novel diagnostic approach to Lynch syndrome and other genetic diseases.

## Results

### Saturation mutagenesis and thermodynamic stability predictions

**Fig 1:**
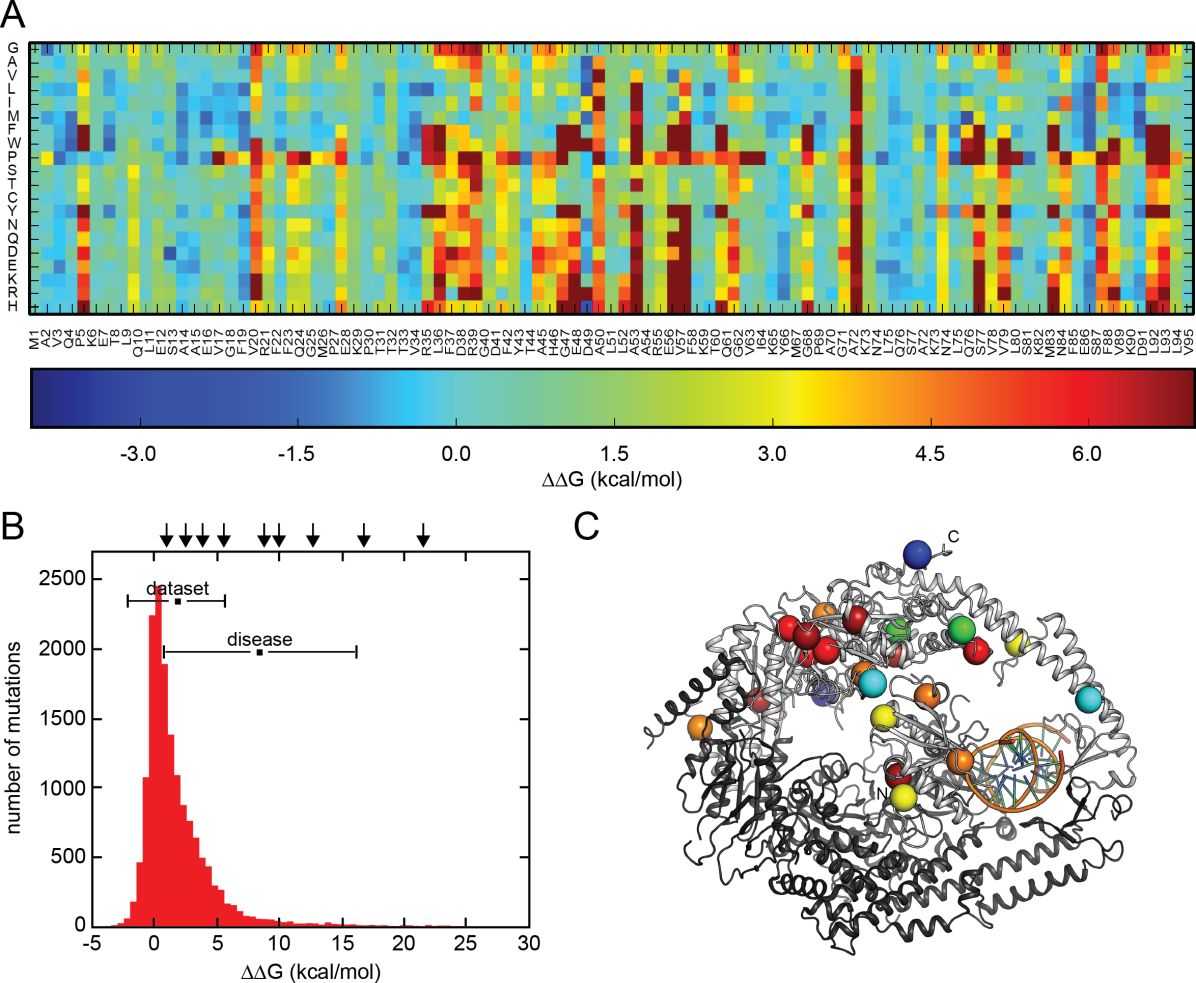
*In silico* saturation mutagenesis and thermodynamic stability of MSH2 mutants. (A) Example of structure-based MSH2 saturation mutagenesis energy calculations shown as a heat-map that represents the change in thermodynamic stability relative to the wild type protein. Due to space limitations, the results are only shown for the first 95 residues (the full dataset is provided in the supporting information S1 File & S2 File). The wild type MSH2 sequence is given below, while the 20 possible residues at each position are shown on the vertical axis. The color bar indicates the magnitude of the change in stability, so that stabilizing mutations are shown in blue, neutral mutations in turquoise, and highly destabilizing mutations in red (mutations that decrease the stability by more than 7 kcal/mol are represented by the same color). (B) A histogram of the number of mutants (vertical axis) plotted against the predicted thermodynamic stability (ΔΔG) (horizontal axis). The mean and variance of the entire dataset, and known disease-linked variants [7] are included in the insert. The arrows mark the ΔΔG values for the set of disease-linked MSH2 mutants included in this study. (C) The positions of 24 selected MSH2 mutants are marked in colors according to the heat-map in (A) on a trace of the MSH2-MSH6 structure (PDB: 2O8E). MSH2 is shown in light gray, MSH6 in dark grey and DNA in orange.

Missense mutations in MSH2 and other mismatch repair proteins have been linked to the hereditary cancer predisposition disorder, known as Lynch syndrome. Obviously, missense mutations may ablate protein function e.g. by mutation in an active site, but also because in general missense proteins are less structurally stable than the wild type protein [10]. To study such stability effects for MSH2, we employed structure-based energy calculations to predict the effects of mutations in MSH2 on the structural (thermodynamic) stability. As a starting point, we used the published crystal structure of the human MSH2-MSH6 heterodimer [11] to perform *in silico* saturation mutagenesis, introducing all possible single site amino acid substitutions into the wild type human MSH2 sequence. We then used two of the most established and tested energy functions for large-scale biophysical modeling, FoldX [12] and Rosetta [13,14], to predict the change in thermodynamic folding stability with respect to the wild type protein (ΔΔG) (Supporting information S1 File, S2 File and S3 File). Both energy functions provide a quantitative description of the inter- and intramolecular interactions that stabilize proteins, and have been extensively benchmarked for ΔΔG prediction over a set of test proteins, with accuracies of about 0.8 kcal/mol [12] and 0.7 kcal/mol [13], respectively. The results presented here are mostly based on our FoldX calculations, but as described further below, calculations using Rosetta gave very similar results. The calculated values report on the change in structural stability of the MSH2-MSH6 complexes, such that negative values indicate variants that are more stable than the wild type, while positive values indicate that the variants are less stable than the wild type MSH2 protein. Thus, mutant variants with ΔΔG>0 have, compared to the wild type sequence, a higher population of (partially) unfolded structures that are prone to misfold or aggregate. Our dataset comprises 19 (amino acids, not including the wild type residue) * 855 (resolved residues in the crystal structure) = 16,245 MSH2 variants. Heat map representations of all the FoldX calculations are included in the supporting information (Supporting information S2 File) and, for the first 95 residues, shown here (Fig 1A). From these data, it is clear that mutations at some positions are tolerated (blue/turquoise vertical columns, e.g. S13), while for other positions most mutations are predicted to destabilize the structure (red vertical columns, e.g. A50). In addition, the structural constraints typically induced by substituting with proline are also evident (red horizontal line for P).

The resulting distribution of ΔΔG values is similar to those described previously for other proteins [10,15] and reveal that most MSH2 mutations only moderately affect MSH2’s thermodynamic stability, i.e. that many ΔΔG values are relatively close to 0 kcal/mol (Fig 1B). Few mutations appear to stabilize MSH2 (e.g. 5% have ΔΔG < -1 kcal/mol), while more are predicted to structurally destabilize MSH2 (e.g. 9% have ΔΔG > 5 kcal/mol). The known disease-causing mutations generally appear to display higher ΔΔG values (mean ΔΔG is 9 kcal/mol for the cancer predisposing variants compared to an average of 2 kcal/mol over all mutants) and are therefore likely to structurally destabilize the MSH2 protein (Fig 1B). Intriguingly, however, some Lynch syndrome-linked MSH2 mutations are predicted to have only a minor effect on protein stability (Table 1; Supporting information S3 File), suggesting a more complex relationship between mutations and disease.

**Table 1:** Stability and function of selected MSH2 mutant proteins.

Selected mutations	FoldX ΔΔG (kcal/mol)	Solvent accessibility (%)	Half-life (hours)	Nuclear localization	MSH6 interaction	Found in patients	Temperature sensitive
Wild type	0.0	-	19±3	Yes	Yes	No	No
R39E	3.4	17.4	4±2	Yes	Yes	No	Ts^a^
A54Y	21.2	0.0	8±0.1	Yes	Yes	No	Ts
L75K	3.0	6.1	5±2	Yes	Yes	No	Ts
Y98C	2.5	19.8	13±2	Yes	Yes	Yes	(Ts)
L135Y	3.4	16.2	22±4	Yes	Yes	No	No
D180F	16.5	0.1	6±1	Yes	Yes	Yes	No
L187P	8.8	0.7	5±1	Yes	No	Yes	No
C199R	5.5	0.0	5±1	(Yes)	No	Yes	No
K228E	0.2	100	20±1	Yes	Yes	No	No
M253Y	-0.1	94.4	20±2	Yes	Yes	No	No
A266W	39.7	0.0	4±0.4	Yes	Weak	No	No
D283K	0.5	100	8±1	Yes	Yes	No	No
C333Y	21.7	0.3	5±0.4	(Yes)	Weak	Yes	No
A399K	9.4	0.3	11±1	Yes	Yes	No	No
D459I	2.4	20.6	13±1	Yes	Yes	No	No
E561V	0.0	78	20±5	Yes	Yes	No	No
D603N	1.0	3.8	6±2	(Yes)	Yes	Yes	No
P622T	3.7	0.0	10±2	(Yes)	Yes	Yes	Cs^b^
A649F	-0.3	100	16±3	Yes	Yes	No	No
G669D	12.7	10.3	7±1	Yes	Yes	Yes	No
G683R	9.8	0.0	7±3	(Yes)	No	Yes	No
P696F	13.8	0.0	4±1	Yes	Weak	No	No
S743P	7.5	0.0	7±4	Yes	Yes	No	No
L851P	5.1	35.3	16±1	Yes	Yes	No	No

a: ts, temperature sensitive. b: cs, cold sensitive. The brackets indicate partial nuclear localization or weak ts phenotype.

### MSH2 mutations lead to reduced steady-state protein levels

Previous studies have shown that the steady-state level of certain disease-linked MSH2 variants is reduced [16,17]. To test this in a more general manner, we selected 24 different missense MSH2 mutants with predicted ΔΔGs spanning from -0.3 to 39.7 kcal/mol for further studies (Table 1). When selecting these, we ensured that the mutations were scattered evenly throughout the MSH2 structure (Fig 1C). To ensure that our observations did not depend on the potential special nature of pathogenic mutations, we included mutations that have been linked to Lynch syndrome and others that to our knowledge have not. Data and known clinical relevance for each of the selected variants are summarized in Table 1.

**Fig 2:**
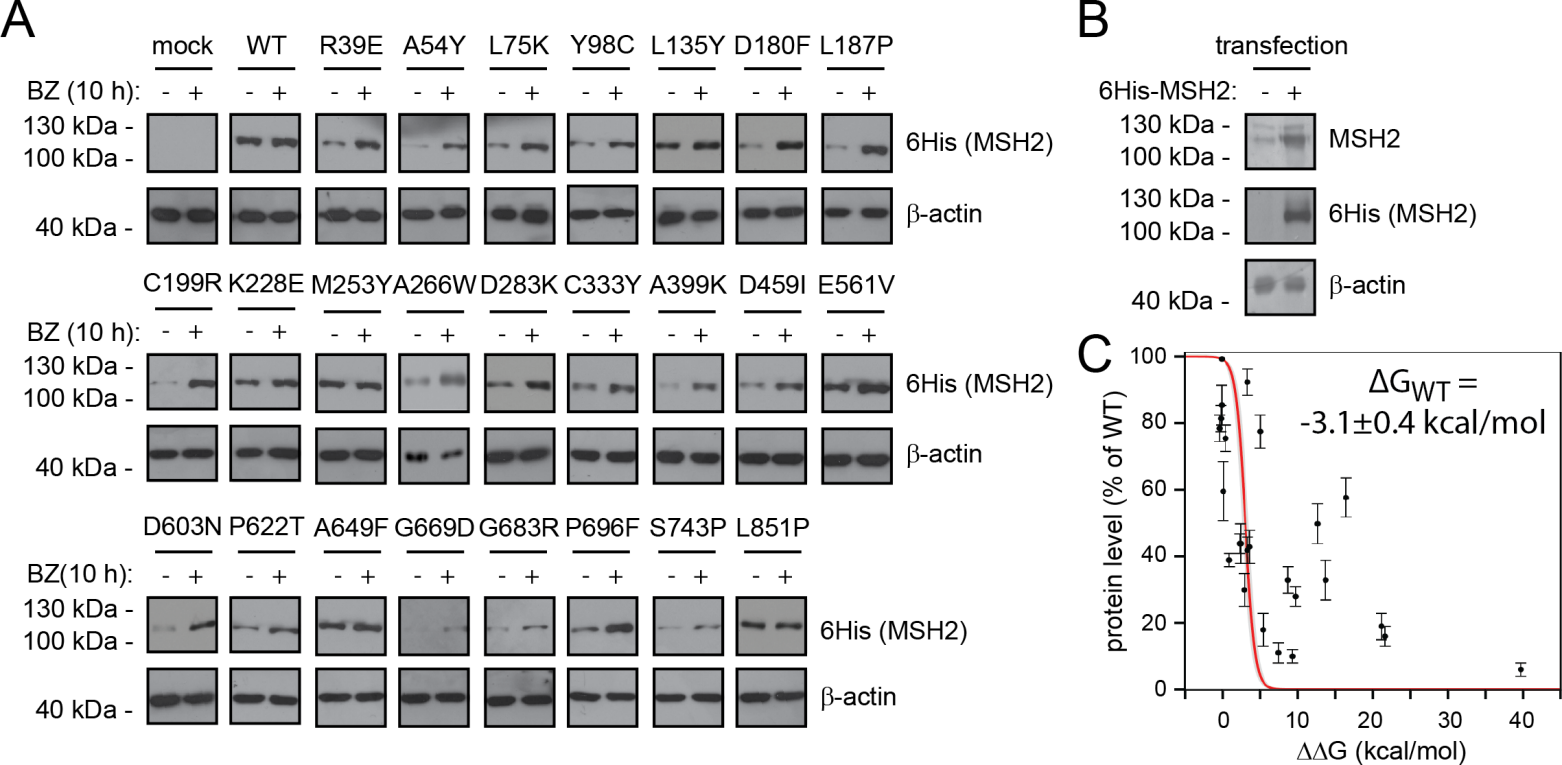
MSH2 mutation leads to reduced steady-state levels due to proteasomal degradation. (A) The steady-state level of the indicated wild type (WT) or MSH2 mutants expressed in U2OS cells was determined using SDS-PAGE and Western blotting with antibodies to 6His-tag on MSH2 in cultures that were either untreated or treated with the proteasome inhibitor bortezomib (BZ) for 10 hours. β-actin served as a loading control. See also supporting information S1 Fig. (B) Blotting, using antibodies to MSH2, revealed that transfection of wild type 6His-MSH2 led to an overexpression of approximately 4 fold. (C) Steady-state levels of the selected MSH2 variants plotted vs. the calculated ΔΔG values. The error bars indicate S.E.M. (n = 3). The red line corresponds to the fit with the thermodynamic model. The grey line indicates the 25th and 75th percentiles after a bootstrapping procedure, in which we fit random subsets of the data to the thermodynamic model (percentiles taken after 5000 iterations of fitting). The ΔG(WT) estimate and error are derived from the same bootstrapping procedure.

The selected point mutants were introduced in U2OS cells and expressed with an N-terminal 6His-tag. Several of the variants had strongly reduced steady-state levels (Fig 2A). When we treated the cells with the proteasome inhibitor bortezomib (BZ), we observed substantially higher steady-state levels, suggesting that the reduced levels are caused by proteasomal degradation of the MSH2 variants (Fig 2A). This effect was not a result of introducing the 6His-tag, since CFP-tagged MSH2 variants were also unstable (Supporting information S1A Fig). In addition, the observed destabilization was also valid for other disease-causing MSH2 variants listed in the OMIM database, such as R524P, P622L, A636P, H639Y and G669V. In contrast, the G322D variant that is also present in OMIM, appeared stable (S1B Fig). Accordingly, previous studies have shown this variant to be benign [18]. As it is also found at a high frequency in the Exome Aggregation Consortium (ExAC) database [19], we suggest that this variant is indeed non-pathogenic, which further strengthens our finding that in general the disease-linked MSH2 variants are structurally more destabilized than non-pathogenic sequence variation (see also below).

Recently, it was proposed that when wild type MSH2 is produced in excess of its binding partners, MSH3 and MSH6, it is ubiquitylated by the histone deacetylase HDAC6 and degraded by the proteasome [20]. Indeed, the MSH2 proteins, produced here, are overexpressed (Fig 2B). We did, however, not observe any change in wild type MSH2 levels upon treatment with proteasome inhibitors, suggesting that the overexpressed wild type MSH2 is not rapidly turned over. Moreover, since knock-down of HDAC6 did not affect the steady-state level of wild type MSH2 or any of the selected MSH2 mutants (Supporting information S1B Fig), we conclude that the reported HDAC6-dependent turnover of orphan MSH2 is not relevant for the MSH2 quality control mechanism that we describe here.

When examining the steady-state level of the MSH2 variants relative to wild type MSH2, we observed that those variants with high ΔΔG values displayed a reduced steady-state level (Fig 2C). However, some MSH2 variants predicted to be structurally rather stable (low ΔΔG values) exhibit low steady-state levels (Fig 2C), showing that the PQC is highly sensitive to abnormal proteins.

The thermodynamic stability (ΔG value) of each mutant can be calculated as the sum of the stability of the wild type protein (ΔG_WT_) and the difference in stability between the wild type and mutant (ΔΔG). While FoldX can predict the latter, ΔG_WT_ remains unknown. We can, however, estimate an effective value of ΔG_WT_ from the data under the rough assumption that the steady-state level in the cell is proportional to the fraction of folded protein ([Fold]/([Unf]+[Fold])), which we in turn can determine from the relationship ΔG_Mut_ = ΔG_WT_+ΔΔG = -RT ln([Unf]/[Fold]). Fitting the data to this relationship results in ΔG_WT_ ~ -3.1 ± 0.4 kcal mol^-1^ (Fig 2C). We note that this value is an estimate of the *effective* stability of MSH2 inside the cellular environment and in the presence of the PQC system, and might potentially differ from the absolute stability of MSH2/MSH6 *in vitro*. The value obtained is also in line with a visual analysis of the data, which suggests a general drop in protein levels for mutants that have ΔΔG > 3 kcal/mol, and an independent estimate obtained using functional studies of MSH2 mutants (see below). We note, however, also the substantial scatter of the data around the fitted line. These deviations may for example be due to inaccuracies in the ΔΔG predictions, differences between *in silico* and cellular stabilities and the specific mechanisms by which the PQC recognizes misfolded proteins. We conclude that a destabilization of roughly 3 kcal/mol is sufficient to cause degradation.

### MSH2 variants are rapidly degraded

**Fig 3:**
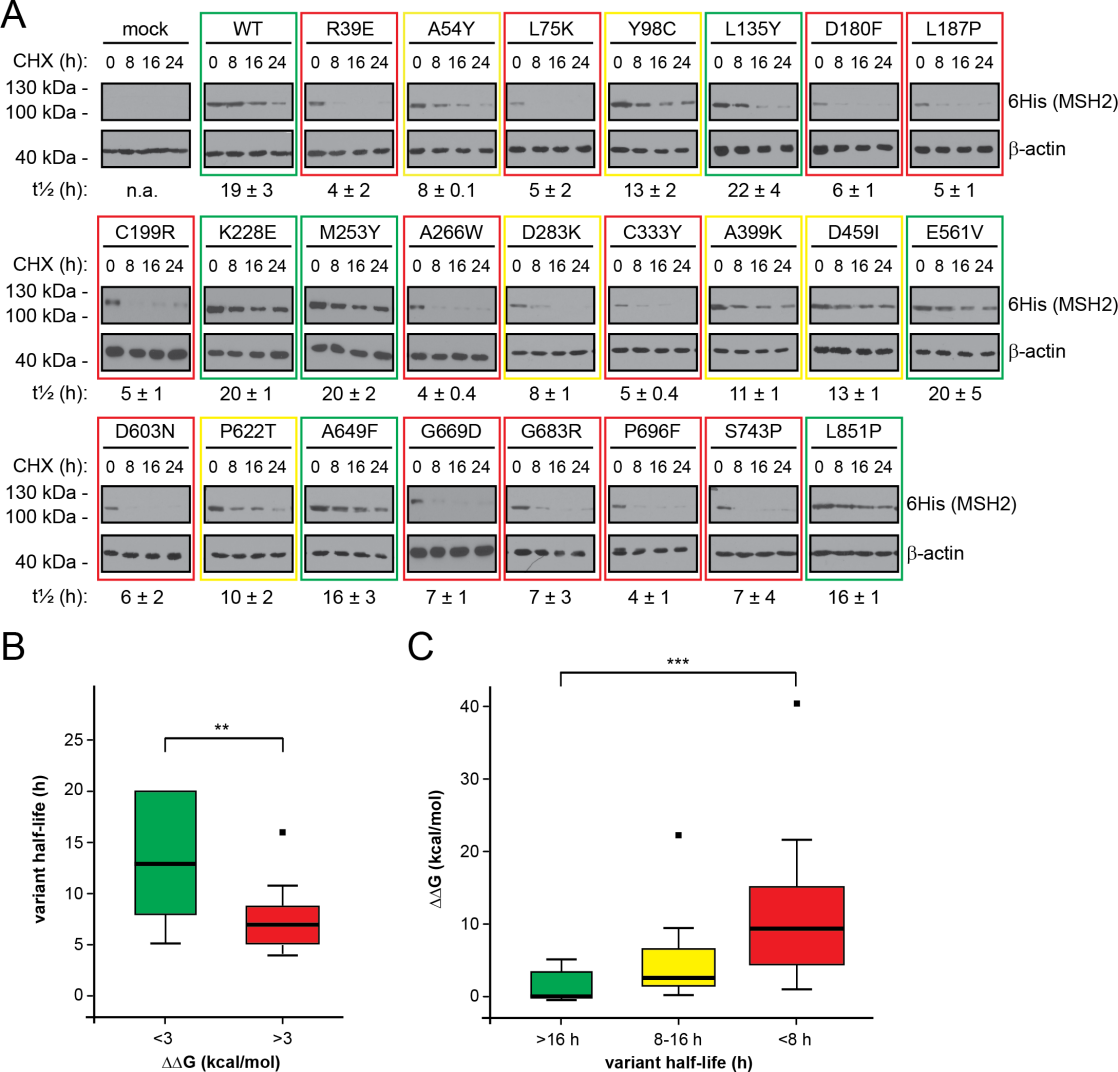
Degradation of MSH2 variants. (A) The degradation of wild type (WT) MSH2 or the indicated MSH2 mutants in U2OS cells was followed at 37°C in cultures treated with cycloheximide (CHX) for 0, 8, 16 and 24 hours. β-actin served as a loading control. The half-life (t½) of each variant was determined by densitometry of this and longer exposures and is given below along with the standard deviation. As a guide, slowly degraded variants have been boxed in green, rapidly degraded variants in red, while intermediate variants are boxed in yellow. See also supporting information S2 Fig. (B) The half-life for the MSH2 variants plotted vs. the calculated ΔΔG values in categories of < 3 kcal/mol and > 3kacl/mol. Bootstrapping shows that average half-life values for low-ΔΔG variants are higher than for high-ΔΔG variants with a p-value of 0.01 (**). (C) A plot of the calculated thermodynamic stabilities of the mutants (vertical axis) against the degradation rate. The horizontal line marks the median and the bars indicate the spread of the data points. *** indicates p < 0.001.

Next, we analyzed if the reduced steady-state levels of the mutant MSH2 variants were indeed caused by rapid degradation. In cultures treated with the translation inhibitor, cycloheximide (CHX), we followed the amounts of MSH2 by Western blotting. We found that wild type MSH2 was relatively stable with a half-life of 19 ± 3 hours (Fig 3A) while those mutant proteins that displayed a reduced steady-state level were rapidly degraded (Fig 3A) (Table 1). Since certain DNA repair components are degraded as part of their normal function [21], we also followed the degradation of wild type MSH2 in cultures treated with the alkylating agent, methylnitronitrosoguanidine (MNNG). However, since MNNG treatment did not affect MSH2 stability (Supporting information S2A Fig), we conclude that the MSH2 variants are turned over as part of a cellular quality control mechanism and not as consequence of their function in mismatch repair. In addition, the turnover of the MSH2 variants was not a result of the overexpression, since wild type MSH2 was degraded with similar kinetics to that observed for endogenous MSH2 (Supporting information S2B Fig), and cells stably transfected to produce selected MSH2 variants at near endogenous levels (Supporting information S2C Fig) degraded the proteins with kinetics identical to those observed for the overexpressed variants (Supporting information S2D Fig).

In general, the rapidly degraded MSH2 variants carried mutations in residues buried within the MSH2 protein and, interestingly, appear to cluster towards the C-terminal ATPase domain (Table 1). Since mutations in residues that are buried and form many contacts often lead to a greater structural destabilization than mutations in surface residues [10,22], we observed that in general those proteins that are predicted as structurally highly destabilized were also rapidly degraded (Table 1). Accordingly, when plotting the half-lives of the MSH2 variants, we observed that those variants with high ΔΔG values displayed a more rapid degradation (Fig 3B). The correlation between ΔΔG and turnover rate was statistically significant (Fig 3C). However, some variants that were rapidly degraded did not display strongly increased ΔΔG values, which suggests that the over-zealous PQC system targets some MSH2 variants that are structurally stable and perhaps retain function.

### Some MSH2 variants are stabilized at low temperatures

**Fig 4:**
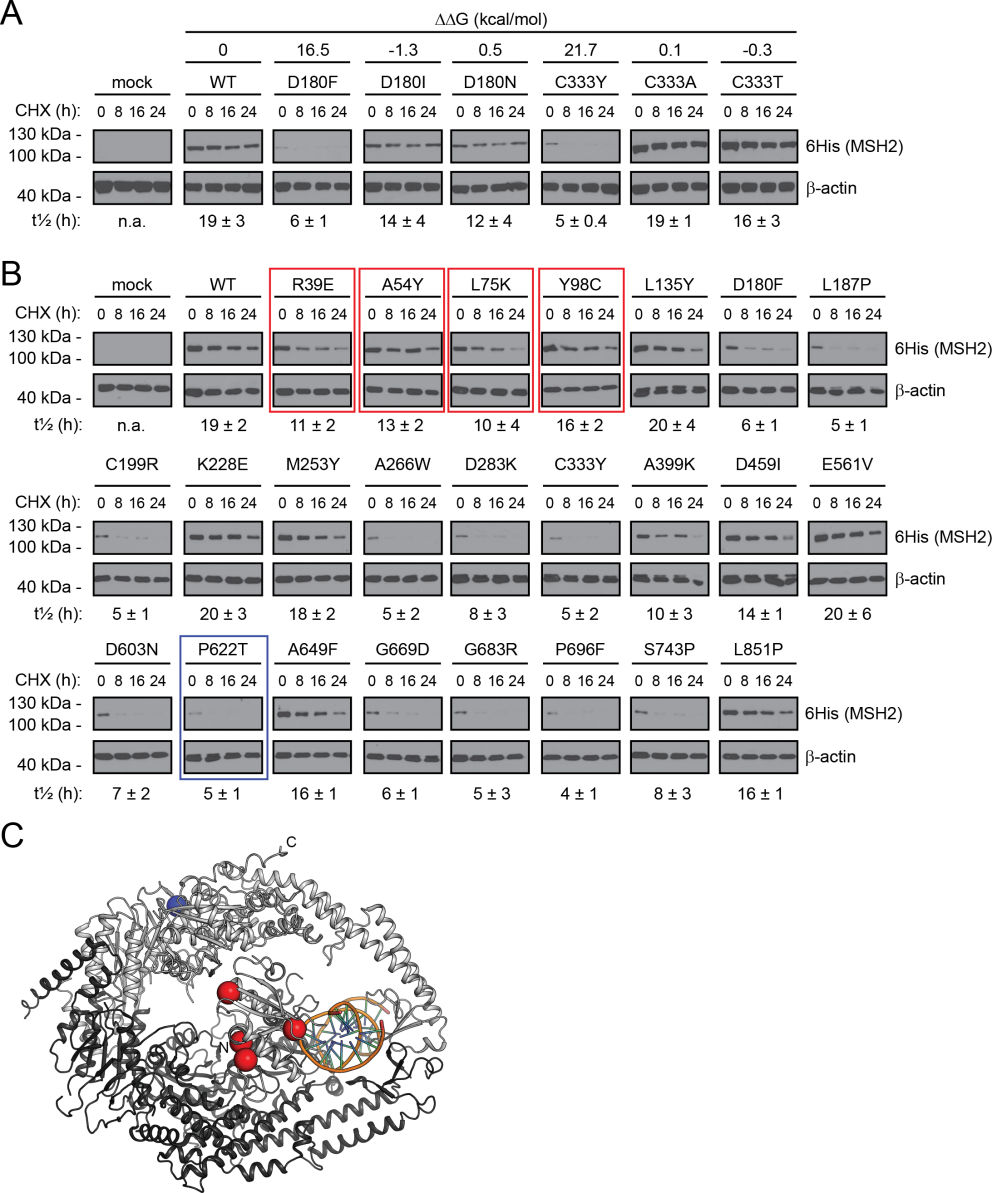
Stabilizing rapidly degraded MSH2 variants by mutation or lowered temperature. (A) The degradation of wild type (WT) MSH2 or the indicated MSH2 mutants in U2OS cells was followed at 37°C in cultures treated with cycloheximide (CHX) for 0, 8, 16 and 24 hours. β-actin served as a loading control. The half-life (t½) of each variant was determined by densitometry of this and longer exposures and is given below along with the standard deviation. Note that MSH2 variants with lower ΔΔG values appear more stable. (B) The degradation of wild type (WT) MSH2 or the indicated MSH2 mutants in U2OS cells was followed at 29°C in cultures treated with cycloheximide (CHX) for 0, 8, 16 and 24 hours. β-actin served as a loading control. Those MSH2 mutants that displayed a temperature dependent degradation are boxed (red, heat sensitive; blue, cold sensitive). The half-life (t½) of each variant was determined by densitometry and is given below along with the standard deviation. See also supporting information S3 Fig. (C) Those MSH2 mutants that displayed a temperature-dependent degradation are marked on a trace of the MSH2-MSH6 structure, red, heat sensitive; blue, cold sensitive. MSH2 is shown in orange, MSH6 in grey and DNA in yellow.

From the experiments above, we conclude that turnover, at least in part, correlates with the predicted thermodynamic stability of the protein. To test this further, we generated another four MSH2 point mutants, exchanging thermodynamically unfavorable residues (high ΔΔG) into more favorable residues (low ΔΔG) at the same position in the protein, and analyzed their degradation as before. Indeed, we found that this dramatically stabilized the MSH2 proteins (Fig 4A). For instance, while the C333Y variant (ΔΔG = 21.7 kcal/mol) is rapidly degraded (t_½_ = 5 ± 0.4 hours), the C333T variant (ΔΔG = -0.3 kcal/mol) is degraded slowly at a rate comparable to wild type (t_½_ = 16 ± 3 hours) (Fig 4A). Thus, it is not just the location of the mutation in the sequence or the structure that is important, but the exact nature of the change in the amino acid side chain chemistry.

In addition to changes in the amino acid sequence, a number of chemical and physical parameters are known to affect the structural stability of proteins. For instance, several misfolded proteins are stabilized at lower temperatures, but some are also destabilized at lower temperatures [23]. To further corroborate the relation between structural protein stability and protein turnover, we repeated the protein degradation assays on the full set of MSH2 variants, but now lowering the temperature from 37°C to 29°C. For some MSH2 variants (R39E, A54Y, L75K) this radically slowed the degradation (Figs 3A and 4B) (Table 1), while one variant (P622T) appeared less stable at 29°C than at 37°C (Table 1). The reduced turnover of the MSH2 R39E, A54Y and L75K variants at 29°C compared to 37°C is not simply a consequence of a reduced UPS activity at lower temperatures, since the turnover of most others variants was entirely unaffected by this change in temperature (Figs 3A and 4B). Also, the cellular amounts of ubiquitin-protein conjugates and proteasomes were unchanged in this temperature interval (Supporting information S3 Fig).

Surprisingly, when we mapped the temperature sensitive mutations onto the MSH2 structure, we found that all clustered towards the MSH2 N-terminal DNA binding region (Fig 4C). Thus, local unfolding of this domain might be particularly temperature dependent rendering these mutations more temperature sensitive. Notably, the corresponding region in the bacterial MSH2 homologue has been shown to be highly dynamic in solution [24].

### The structural stability calculations can predict dimerization with MSH6

Although our data suggest that the thermodynamic stabilities of MSH2 variants is the primary factor that decides their turnover, some of the variants, included in our selection, are rapidly degraded despite having structural stabilities only slightly lower than wild type. For instance, the disease-causing D603N variant is rapidly degraded (t½ = 6 ± 2 hours) while the mutation is not predicted to strongly affect MSH2 structure (ΔΔG = 1.0 kcal/mol). We therefore speculated whether this and other MSH2 variants are still functional, and in this way, similar to other genetic disease such as cystic fibrosis [25], Lynch syndrome could be explained by the over-zealous degradation machinery.

To test this hypothesis, we first analyzed the subcellular localization of the selected MSH2 variants. All variants localized to the nucleus similar to the wild type protein [26], (Supporting information S4A and S4B Fig and Table 1), although the signal intensity varied (Supporting information S4A Fig) as expected, based on the reduced steady-state level.

**Fig 5:**
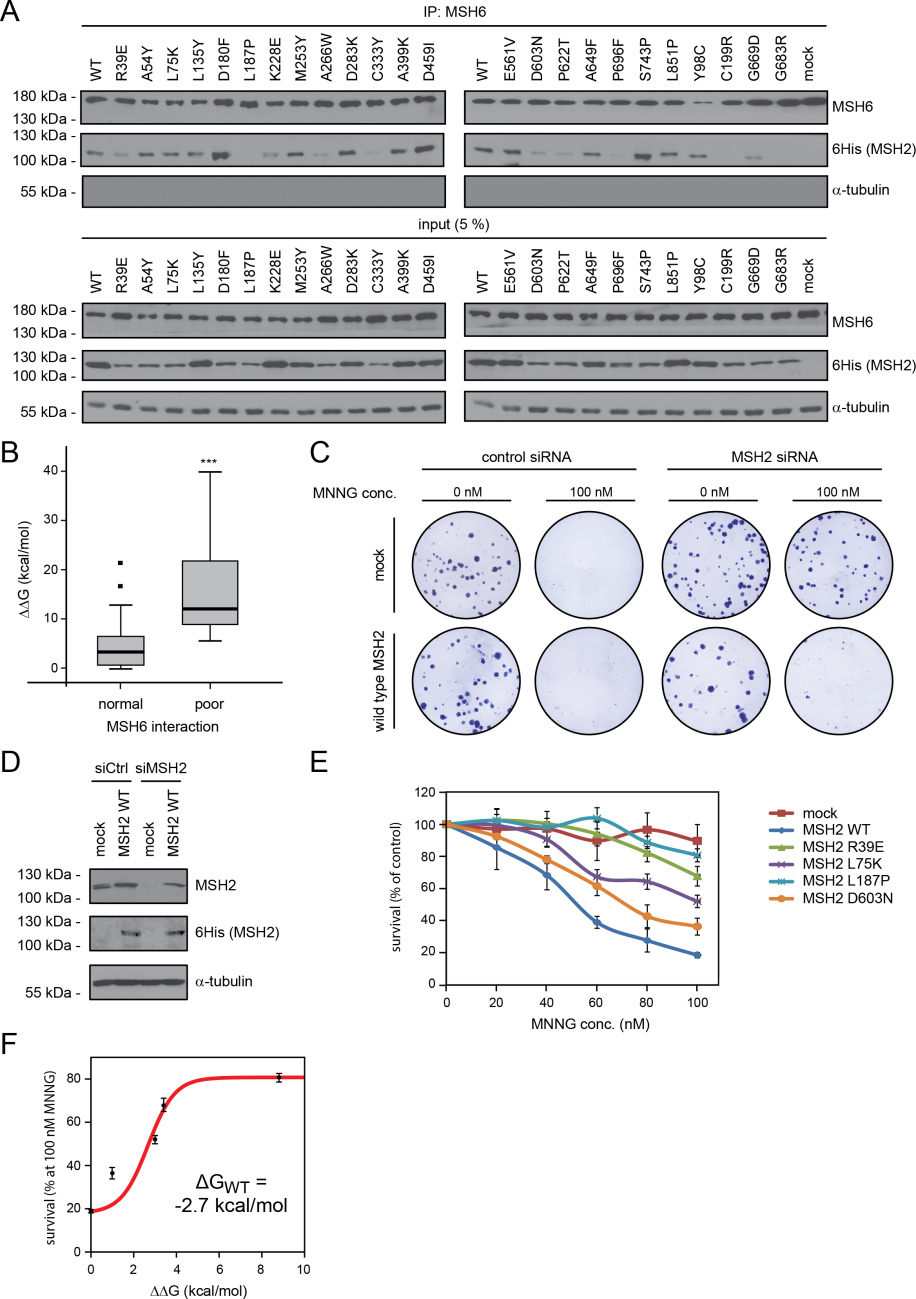
Functional analyses of MSH2 variants. (A) The MSH2 variants were analyzed for interaction with MSH6. Endogenous MSH6 was immunoprecipitated and the precipitated material analyzed by SDS-PAGE and Western blotting using antibodies to the 6His-tag on the MSH2 variants. Input samples (5%) were included as a control. α-tubulin served as a loading control. To obtain sufficient amount of the MSH2 variants the cells were treated with the proteasome inhibitor bortezomib (BZ) for 6 hours before harvest. See also supporting information S4 Fig. (B) The calculated thermodynamic stabilities of the test mutants (vertical axis) are plotted towards categories of normal MSH6 interaction or reduced MSH6 interaction. The horizontal line marks the median and the bars indicate the spread of the data points. *** indicates p < 0.001. (C) U2OS cells stably transfected with either vector (upper row) or wild type 6His-tagged siRNA resistant MSH2 (lower row), were transfected with control siRNA or siRNA to endogenous MSH2 and treated with either 0 or 100 nM MNNG. The surviving colonies were stained with crystal violet. (D) The siRNA-mediated knock-down of endogenous MSH2 is shown by Western blotting using antibodies to MSH2 and the 6His-tag on the recombinant (siRNA-resistant) MSH2. α-tubulin served as a loading control. Note that the recombinant variants are not overexpressed compared to the endogenous MSH2 (E) The survival of the U2OS cells, stably transfected with siRNA to endogenous MSH2, was monitored in response to increasing amounts of the alkylating agent MNNG. Note that cells containing wild type MSH2 fail to survive, whereas the vector control cells survive. The tested MSH2 variants display an intermediate MNNG sensitivity. The error bars indicate the S.E.M. (F) Estimate of the stability of MSH2 assuming that the wild type and MSH2-L187P protein can be used as approximations for fully folded and non-folded protein, respectively. The red line shows the fit to the thermodynamic model.

To better discriminate between functional and dysfunctional MSH2 variants we therefore turned to mapping the interaction partners of wild type MSH2 and of L187P that displays a high ΔΔG value (8.8 kcal/mol), is rapidly degraded and therefore likely to be highly misfolded and not functional. To quantify any differences in terms of protein binding between wild type MSH2 and the L187P variant, a quantitative proteomics experiment was undertaken. All mass spectrometry data are included in the supporting information (Supporting information S4 File). Affinity purification was used to purify proteins from cells treated with bortezomib (to ensure that L187P was not degraded) expressing vector (control), 6His-MSH2 (wild type) or 6His-MSH2-L187P (Supporting information S5A Fig) in quadruplicates. The purified proteins were digested with trypsin, and LC-MS/MS in combination with MaxQuant data analysis was used for identification and quantification. Label-free intensities were converted to ratios by comparison of the 6His-MSH2 purification protein intensities with those derived from the control purifications. MSH2, MSH3 and MSH6 were the three most enriched proteins in 6His-wild-type MSH2 preparations (Supporting information S5B Fig). In the presence of proteasome inhibitors L187P was expressed at roughly half the level of the wild type (Supporting information S5A, S5B and S5C Fig). MSH6 was almost 8 fold reduced in abundance, and MSH3 almost 30 fold reduced in the L187P samples compared to wild type (Supporting information S5B and S5C Fig). The reduced binding of L187P to MSH3 and MSH6 was confirmed independently by Western blotting (Supporting information S5D Fig). A wider screen showed that wild type MSH2 and several MSH2 variants co-precipitated with endogenous MSH6 (Fig 5A). However, some MSH2 variants displayed only weak interactions with MSH6 (e.g. P696F) and yet others appeared completely inept at MSH6 binding (e.g. G683R). Variants that interact with MSH6 had an average loss of stability of 5 ± 1 kcal/mol (mean ± SEM) while those that interacted poorly with MSH6 were more structurally unstable (15 ± 6 kcal/mol; p < 0.001) (Fig 5B). We conclude that the structural stability calculations allow prediction of MSH2-MSH6 dimerization potential.

### The thermodynamic stability correlates with residual protein function

We next performed experiments to see whether certain unstable MSH2 variants would at least retain some function. A characteristic feature of MSH2 loss-of-function and Lynch syndrome cancers is increased resistance to DNA damage [27]. Accordingly, we found that siRNA-mediated knockdown of endogenous MSH2 rendered U2OS cells resistant to an otherwise lethal dosage of the alkylating agent, MNNG (Fig 5C). Stable introduction of siRNA resistant, wild type MSH2 did not lead to appreciable overexpression (Fig 5D), but re-established the MNNG sensitivity (Fig 5C). The tested MSH2 variants appeared partially sensitive (Fig 5E). Indeed, we found a strong correlation (*r*^2^ = 0.81; p = 0.02) between the predicted loss of stability (ΔΔG) and the ability to grow in the presence of MNNG (Fig 5E), suggesting that the assay is able to probe the amount of functional MSH2. Even at the highest MNNG concentration (100 nM) the L187P variant was not statistically different from the vector control. This is in agreement with the interaction data, and indicates that L187P is misfolded to an extent where it has lost all activity.

The correlation between the change in stability and resistance to MNNG offers us an opportunity to provide an independent estimate of the stability of MSH2 (ΔG_WT_). Assuming that the wild type protein is fully folded and L187P is mostly unfolded, we can fit the observed activities (percent survival at 100 nM MNNG) to estimate ΔG_WT_ (Fig 5F). The value obtained (-2.7 kcal/mol) is in good agreement with the independent estimate obtained from the intracellular protein levels (-3.1 kcal/mol, Fig 2C), and again corresponds visually also to the magnitude of destabilization that is needed to see a substantial difference from the wild type protein. Although both estimates are associated with uncertainty, their agreement lends additional credibility to the values and suggests a relatively low effective stability of MSH2 in the cell that, as demonstrated above, also includes effects from interactions with partner proteins. We note also that the general agreement between the effect on steady-state levels and residual activity also suggests that loss of stability is a major factor leading to loss-of-function for these variants.

### Using thermodynamic stability calculations to predict pathogenic mutations

**Fig 6:**
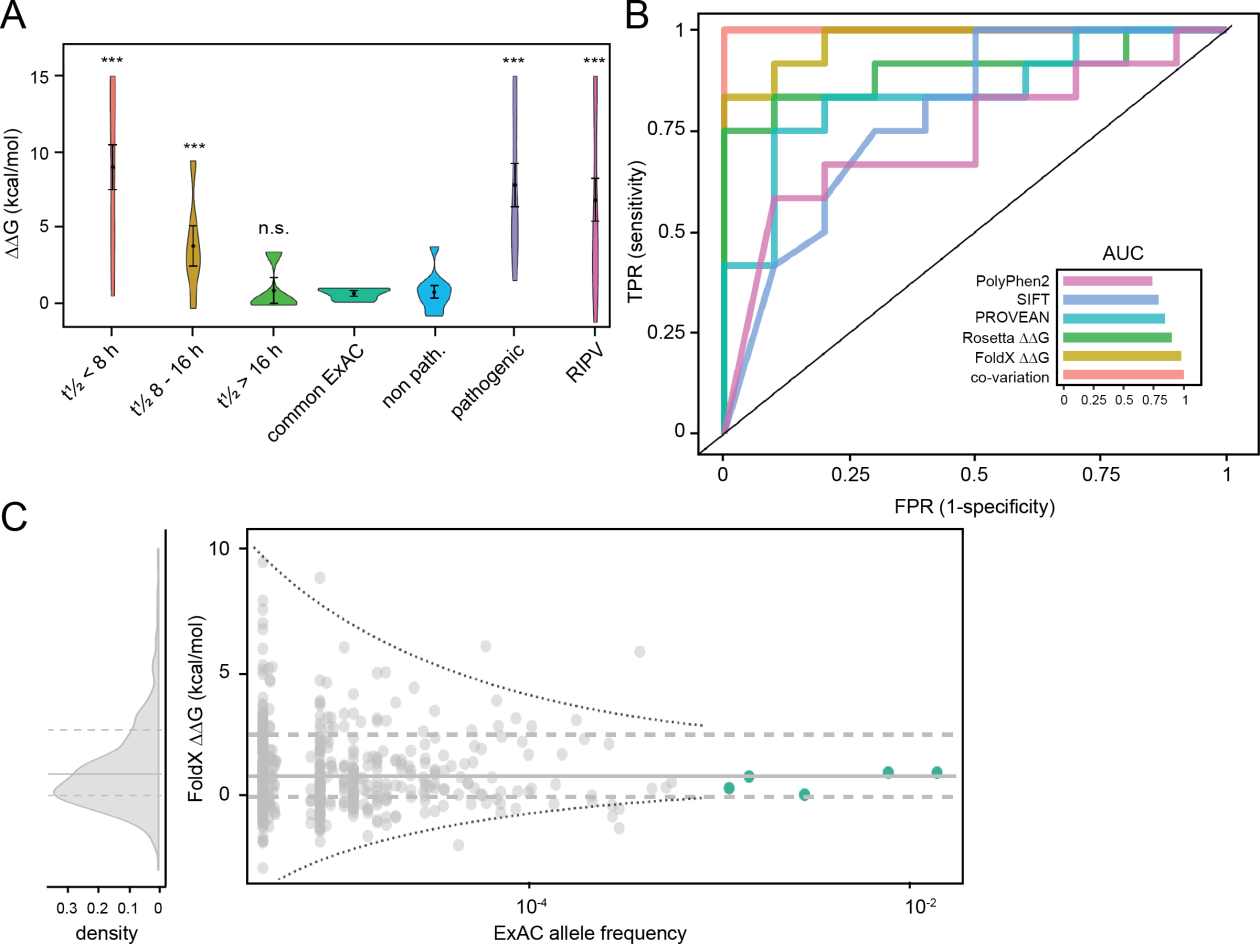
Pathogenicity predictions of *MSH2* mutations. (A) Distributions of FoldX ΔΔG scores for MSH2 variants tested in this work with short (red), intermediate (yellow), and long (light green) half-life (t½), common variants found in ExAC (dark green), known non-pathogenic (blue) and pathogenic (purple) variants, and recently identified pathogenic variants (RIPV, magenta) [30]. Dots indicate the mean ΔΔG score for each group, and the bars indicate the standard error of the mean. (B) Receiver operating characteristics (ROC) curves for the selected predictors of MSH2 variant pathogenicity: co-variation (red), FoldX ΔΔG (yellow), Rosetta ΔΔG (green), PROVEAN (cyan), SIFT (blue), and PolyPhen2 (purple). Accuracy is assessed on known pathogenic and non-pathogenic variants according to Houlleberghs *et al*., 2016. As the area under the curve (AUC) indicates, while all predictors show reasonable performance, ΔΔG and co-variation provide considerably higher accuracy. (C) The distribution of FoldX ΔΔG scores vs. the allele frequency of MSH2 mutations found in the Exome Aggregation Consortium (ExAC) database. Note that alleles that are found at a high frequency in the population, and are therefore unlikely to be pathogenic, appear stable (display low ΔΔG values). The three horizontal lines correspond to quartiles. For further information, refer to S6 Fig.

An advantage of the structural stability calculations on missense variants that we present here is that it may potentially bypass laborious laboratory testing and immediately provide a clinical geneticist with an estimate of whether a particular MSH2 missense variant is pathogenic. The currently employed clinical tools (e.g. CADD, SIFT, PolyPhen2, PROVEAN) provide sequence-based predictions of whether a mutation is likely to be pathogenic [28–31]. While sequence-based predictors have the clear advantage of being technically applicable to virtually all proteins, the structural calculations utilize atomic details and thus allows not only more accurate predictions, but may also enable mechanistic insights (e.g. our observation on ts mutations in the DNA binding domain above) [32,33]. ΔΔG values for variants with half-life > 16 h are significantly lower than those for variants with a half-life < 8 h (Fig 6A). Importantly, the biophysical calculations are able to separate the group of moderately stable proteins (half-life 8–16 h, Fig 6A), while the sequence-based predictors we tested considered those variants as equally pathogenic as the rapidly degraded variants (Supporting information S6 Fig). To compare more directly FoldX and the four sequence-based methods for their ability to distinguish known pathogenic and non-pathogenic variants [34], we repeated these calculations for CADD, SIFT, PolyPhen2, PROVEAN also (Supporting information S6 Fig). Interestingly, we found that while all methods perform reasonably well, FoldX is substantially better at distinguishing pathogenic from non-pathogenic variants (Fig 6B and Supporting information S6 Fig), demonstrating the potential power of structure-based methods.

To corroborate our studies on MNNG sensitivity suggesting that the structural stability of MSH2 variants correlate with MSH2 function, we analyzed the recent data of Houlleberghs and co-workers [34]. ΔΔG values for the majority of the variants reported to be pathogenic by Houlleberghs *et al*. are > 3 kcal/mol and would thus also have been predicted to be pathogenic from the biophysical calculation (Fig 6A).

As a separate method for predicting the biological consequences of mutations, we also turned to more detailed analyses of a multiple sequence alignment of MSH2 homologues. In particular, we created a statistical model of such an alignment that both takes residue conservation into account, but also the non-trivial couplings that occur as a consequence of amino acid co-variation (see Materials and Methods section). Such calculations are known to provide accurate predictions of changes in stability [35] and have recently been used to assess pathogenicity [36,37]. In contrast to the structure-based calculations, in which we examine whether loss of stability is correlated with disease, these calculations do not assume or provide direct insight into the molecular mechanisms that underlie the disease-causing variants.

To quantify the ability of FoldX, Rosetta, co-variation, and the more established sequence-based methods (CADD, SIFT, PolyPhen2 and PROVEAN) to distinguish known pathogenic and non-pathogenic variants [34], we used all these methods to assess the impact of known neutral and Lynch-syndrome-causing mutations (Supporting information S6 Fig). In particular, we performed a “receiver-operating characteristic” analysis in which we compare the different methods’ ability to separate the two classes of mutations. Interestingly, we found that while all methods perform reasonably well, the biophysical calculations (FoldX, Rosetta) and co-variation are substantially better at distinguishing pathogenic from non-pathogenic variants (Fig 6B and Supporting information S6 Fig), demonstrating the potential power of structure-based methods. In line with recent findings [37] we also find that the co-variation analysis increases the predictive power over a simpler conservation analysis (Supporting information S6 Fig).

Finally, we analyzed the predicted protein stabilities of *MSH2* missense mutations found the Exome Aggregation Consortium (ExAC) database [38]. Indeed this revealed that those MSH2 variants that are found at a high frequency in the population, and therefore likely to be benign, all display low ΔΔG values, indicating that these MSH2 proteins are stable and functional (Fig 6C). We note also that while a few more destabilizing mutations are found with much lower frequencies, we cannot assess whether these are due to prediction noise, whether these individuals have an increased risk of Lynch syndrome, or whether these individuals have other compensatory mutations in their genomes. Nevertheless, the finding that all common variants are predicted to have little effect on stability supports the observation that computational ΔΔGs can help identify damaging mutations and should eventually be considered for use in clinical practice in addressing the challenging issue of which rare mutations are pathogenic and which are neutral [39].

In conclusion, our results demonstrate that biophysical calculations performed *in silico* can predict the structural stability, function and turnover of proteins in cells, and allow insights into the molecular mechanisms underlying disease. Such methods may therefore after further testing perhaps be applied diagnostically to sort disease-causing missense mutations from harmless genetic variations.

## Discussion

Lynch syndrome is a common autosomal syndrome characterized by early onset neoplastic lesions in a variety of tissues and microsatellite instability caused by heterozygous loss-of-function germline mutations in genes encoding components of the DNA mismatch repair (MMR) system [8]. In eukaryotes, MMR is accomplished by the MutS heterodimers MSH2 and MSH6 or MSH2 and MSH3, which first recognize and bind mismatched base pairs and then recruit downstream repair components [40]. Loss-of-function mutations in these components result in a mutator phenotype, which consequently leads to cancer predisposition. In addition, mismatch repair-defective tumors are often associated with resistance to conventional chemotherapies, including temozolomide, 5-fluoruracil and cisplatin [8].

PQC systems root out abnormal or misfolded proteins [1,2], such as those encoded by missense mutations [5,6]. In general, these systems rely on molecular chaperones to either refold the misfolded proteins or target them for degradation via autophagy or through the UPS [41–47]. Degradation of proteins that are structurally perturbed, but still functional, has been linked to disease, as in cystic fibrosis [25,48] and, as we show here for Lynch syndrome, which should therefore be considered a protein folding disease.

At present, our understanding of what determines whether a misfolded protein is refolded or degraded is limited, though presumably the structural stability of the substrate protein is one crucial determinant. To formally test this requires, however, that biological and thermodynamic stabilities of closely related proteins are determined in parallel. To accomplish this, we chose the MSH2 protein as a model substrate for the following reasons: First, structural data for MSH2 are available [11], thus allowing us to perform accurate thermodynamic stability predictions. Second, the wild type MSH2 protein is stable and is not turned over as part of its normal cellular function. Hence, any degradation that we may observe for MSH2 mutants can be attributed solely to a reduced structural stability. Third, MSH2 is a rather large protein, and is therefore likely to be highly dependent on temperature for correct folding [49], allowing us to use this simple physical parameter to regulate the degree of misfolding.

Previous studies on yeast MSH2 mutants suggest that a high proportion of missense mutations affect the steady-state protein levels [7,50]. Our studies on human MSH2 variants confirm these findings. Out of the 24 MSH2 variants studied here, we found that 18 displayed a lower steady-state level and were more rapidly degraded than the wild type protein. For all those variants, the protein levels could be increased by treating cells with proteasome inhibitor, demonstrating that the turnover occurs via the UPS. As hypothesized, those mutations that were predicted to be highly structurally destabilizing were also scored as being rapidly degraded, whereas other structurally less destabilizing mutations at the same positions slowed protein turnover. These observations, combined with our finding of some variants that display a temperature sensitive degradation (i.e. degraded at 37°C, but stable at 29°C), support that the structural stability of the mutants is a primary determining factor for the degradation. This is further reinforced by our finding that several of the variants that we had scored as highly structurally unstable displayed a strongly reduced MSH6 binding. However, in all cases the structural destabilization inferred by the mutations appeared rather subtle, since all the tested MSH2 variants localized, like wild type MSH2, to the nucleus, and none formed protein aggregates.

Some MSH2 variants were rapidly degraded although the structure-based energy calculations only predicted them to be moderately destabilized. Thus, although we find a clear overall relationship between the predicted change in thermodynamic stability and the cellular protein degradation rates, the details of that relationship are likely more complex. For example, our calculations focus upon the effect of the mutations on the global stability of the protein, but different regions of a protein can differ in local stabilities [24]. Thus, mutations with the same overall destabilization, but located in different regions of the protein structure, might differentially affect local stabilities. A more quantitative analysis would thus require knowledge about any possible local unfolding as well as the mechanisms by which these are recognized by the PQC systems. Our results are also reminiscent of the results of a study on the relationship between destabilization and function in ubiquitin [51]. That study found that core mutations that were only mildly destabilizing were fully functional, whereas mutations with intermediate levels of destabilization had more varied functional effects.

Our finding that MSH2 variants are targets of the cellular PQC system prompts the question as to the upstream components such as chaperones and E3 ubiquitin-protein ligases that target the variants for degradation, and whether blocking degradation would be beneficiary. In yeast cells, MSH2 is targeted by the E3 San1 [7], whereas in mammalian cells orphan wild type MSH2 was proposed as a target for HDAC6-catalyzed ubiquitylation [20]. However, as of yet no mammalian orthologue of San1 has been found [52], and as shown here, we did not observe any change in MSH2 stability upon knock-down of HDAC6. Consequently, the E3 ubiquitin-protein ligase responsible for targeting mutant variants of MSH2 in mammalian cells remains to be identified, and possibly multiple E3s are involved.

With the rapid progress of genome sequencing, it is anticipated that genetic testing will soon become a routine procedure. This will further the implementation of personalized medicine but as a consequence the research community will be faced with the daunting task of sorting disease causing genetic variants from harmless natural variants. Because of the rarity of many pathogenic mutations it may be difficult to rely on purely statistical approaches to solve this problem. To approach this issue, determining the structural stability of proteins *in silico*, like we did here, could provide a powerful diagnostic tool, but may also aid clinicians in differentiating treatment according to the activity of the mutant protein. We stress also that our finding that changes in a specific molecular property (protein stability) correlate with phenotype (disease) suggests strongly a mechanistic model that would be hard to obtain using tradition methods for assessing pathogenicity. To develop this approach to a more clinically relevant tool will, however, require both improved prediction accuracy for stability changes, and an improved understanding of the relative importance of local vs. global unfolding and the mechanisms by which these transiently misfolded structures are recognized by the PQC system. We note, however, that in a direct comparison using known variants with known pathogenicity, our approach outperforms several currently employed methods. Further, our finding that a co-variation approach and the stability predictions both reach roughly the same prediction accuracy suggests that the co-variation calculations, at least in the case of the studied MSH2 variants, capture mostly stability effects. As these two methods have different limitations (e.g. availability of structural information vs. availability of a large number of homologues sequences), we suggest that the two approaches will complement each other well.

Nevertheless there are some outliers, including the rapidly degraded D603N MSH2 variant, which has a relatively low ΔΔG (1.0 kcal/mol), as well as A399K and L851P, which were predicted to be unstable (9.4 and 5.1 kcal/mol, respectively) but found experimentally to have wild-type-like *in vivo* stability. Co-variation and PolyPhen2 both correctly predict the detrimental effect of D603N, indicating that conservation may in some cases be more informative than biophysical changes, especially in cases of similar amino acids. A399K is predicted neutral by co-variation; possibly the mutation could be accommodated by local reorganization, but this remains difficult to model structurally. All methods used here predict L851P to be detrimental, in contrast to experimental findings, underlining the difficulty of such predictions, perhaps especially in flexible regions of the protein. Detailed scores for each predictor and variant can be found in the Supporting Information (Supporting information S3 File).

In conclusion, our results show a logical, but until now unproven, correlation between predicted structural protein instability and protein turnover. In turn this may provide clinicians with a novel tool to score the severity of missense mutations of uncertain clinical significance. In case of Lynch syndrome, this information is highly relevant, since an early diagnosis can strongly increase survival [9].

## Materials and methods

### Buffers

Buffer A: 50 mM Tris/HCl pH 7.4, 150 mM NaCl, 1 mM EDTA, 0.5% NP-40, 1 mM PMSF and Complete protease inhibitors (Roche). Buffer B: 50 mM sodium phosphate pH 7.4, 50 mM NaCl, 0.5% NP-40, 10 mM imidazole, 10 mM β-mercaptoethanol, 1 mM PMSF and Complete protease inhibitors (without EDTA) (Roche). PBS: 10 mM Na_2_HPO_4_, 1.8 mM KH_2_PO_4_, 137 mM NaCl, 3 mM KCl, pH 7.4.

### Plasmids

For expression of MSH2, full-length wild type human MSH2 cDNA was inserted into pcDNA-DEST40 (Invitrogen). An N-terminal RGS6xHis-tag was inserted upstream of an SRS linker peptide, before MSH2 Met1. All point mutants were generated by Genscript. Select MSH2 mutants were cloned into pcDNA5/FRT (Invitrogen) for stable integration into an U2OS cell line harboring FLP recombination sites (kindly provided by Dr. Jakob Nilsson, University of Copenhagen).

### Cell culture

U2OS cells were maintained in Dulbecco’s Modified Eagle medium (DMEM) supplemented with 10% fetal-calf serum (Invitrogen), 5000 IU/mL penicillin, 5 mg/mL streptomycin and 2 mM glutamine at 37°C in a humidified atmosphere containing 5% CO_2_. Stable MSH2 transfectants were generated using the FlpIn system (Invitrogen) using 50 μg/mL Hygromycin B (Invitrogen) and 50 μg/mL Zeocin (Invitrogen) for selection.

### Electrophoresis and blotting

Proteins were separated on 7 cm x 8 cm 8% acrylamide gels and subsequently transferred to 0.2 μm nitrocellulose membranes. Membranes were blocked in PBS containing 5% fat-free milk powder and 0.1% Tween-20. Membranes were then probed with the indicated antibodies overnight.

Antibodies and their sources were: anti-human MSH2 (CalBiochem), anti-human MSH6 (BD Biosciences), anti-human MSH3 (Abcam), anti-RGSHis (Qiagen), anti-HDAC6 (Cell Signaling Technology), anti-tubulin (Sigma) and anti-β-actin (Sigma). All secondary antibodies were purchased from DAKO.

### Transfections

For DNA U2OS cells were transfected with FugeneHD (Promega) at a DNA:FugeneHD ratio of 1:3.5, according to the manufacturer’s instructions. Before transfection, media was replaced with OptiMEM (Life Technologies), and cells were subsequently incubated with the transfection mix for 4 hours. For transfection in 6-well plates, 1 μg of DNA pr. well was used and scaled accordingly for different well sizes.

For siRNA U2OS cells were reverse transfected with 50 nM On-target Smart Pool siRNA to MSH2 (Dharmacon) using Lipofectamine RNAiMAX (Invitrogen). The mix was then added to newly-seeded cells and medium replaced with complete DMEM after 24 hours. Experiments were performed 48 to 72 hours after transfection.

### Co-precipitation experiments

For binding studies a confluent dish of transfected U2OS cells was lysed in 600 μL buffer A on ice. Cell extracts were cleared by centrifugation at 15000 g for 20 minutes at 4°C and 30 μL and was taken for input. The remaining lysate was then incubated with 5 μL of MSH6 antibody (BD Biosciences) for 2 hours at 4°C before adding Protein G Sepharose (GE Healthcare). After further incubation for 2 hours at 4°C under gentle agitation, the beads were washed by centrifugation 4 times in buffer A. Bound proteins were eluted in SDS sample buffer.

### Protein degradation experiments

The degradation of proteins was followed in cultures incubated with 10 μg/mL cycloheximide in serum-free DMEM. Bortezomib (LC Laboratories) was used at 25 μM.

### Cell survival assays

Stable cell lines were reverse transfected with siRNA against endogenous MSH2 according to the above protocol. Approximately 72 hours after transfection, cells were trypsinized, counted, and seeded into 6-well plates at 500 cells/well. After 24 hours, the media was replaced with 2 mL complete DMEM containing 20 μM O^6^-BG (Sigma) for 1 hour. Subsequently, 1 mL of complete DMEM containing 3x the desired final concentration of MMNG (Sigma) was added to the 2 mL O^6^-BG-containing media. After 9 days the cells were stained with crystal violet and the colonies were counted.

### *In silico* saturation mutagenesis with FoldX

The FoldX energy function version 3.1 was used to estimate the free-energy change upon mutations of MSH2 [12]. FoldX calculations were carried out for each of the monomer structures included in the PDB entry 2O8E [11] to assess the reproducibility of the results, and the average is reported here. The RepairPDB function of FoldX was first applied to the wild type structures. Structures for saturation mutagenesis were generated using an in-house Python program that allows for the introduction of all 19 possible point mutants at each position of the protein using multithreading calculations. The BuildModel function of FoldX was employed and five independent runs were carried out and then averaged. The typical prediction error of FoldX is about 0.8 kcal/mol [53].

### *In silico* saturation mutagenesis with Rosetta

We used the Rosetta version with GitHub SHA-1 6922a68c56c0a3c5f64570c55097ba5d5439e22c (Nov 2016) with the ΔΔG protocol 13 with local optimization [13] and the Talaris2014 energy function [54]. Calculations are based on a minimized structure of the PDB entry 2O8E, as described in the published ΔΔG protocol [13], and constrained not to diverge more than 0.5 Å from the backbone in the crystal structure. The average energy of the lowest 3 out of 15 models for each possible mutation is reported here.

### Co-variation-based prediction of pathogenic mutations

As an additional method for predicting pathogenicity of missense mutations, we turned to the statistical analysis of an MSH2 multiple sequence alignment. More specifically, we built a global statistical model [55] of the multiple sequence alignment to predict the likelihood of any given MSH2 sequence. To emulate a change in thermodynamic stability of a given mutant, we calculate the difference in log-likelihood between the wild-type and the mutant variant. This global statistical approach, as opposed to local, allows us to exploit all sequence constraints encoded by evolution, regardless of availability of high-resolution structural data or putative molecular mechanism. Thus, for example, residues critical for interaction interfaces will score similar to destabilizing mutations or changes to catalytic residues. Similar methods are known to provide accurate predictions of ΔΔG [35] and have recently been applied successfully to identify a range of known disease-related mutations [36].

### Imaging

Cell imaging was performed in thin-bottomed black 96-well plates. Microscopy was performed capturing 12 fields/well in an InCell2200 (GE Healthcare). Filters were DAPI (ex 390 nm, em 432 nm) and TexasRed (ex 575 nm, em 620 nm). Images were analyzed with the InCell Developer Toolbox (GE Healthcare). Nuclear segmentation and cellular segmentation were obtained using “object segmentation” and non-transfected cells were excluded, based on the intensity levels in the non-transfected controls. To analyze the fraction of intensity in the nucleus, the total intensity of the red channel in each cell was measured as well as the total intensity of the nuclear area in the red channel, and the ratio between the two determined.

### Mass spectrometry

For mass spectrometry, stable U2OS cell lines expressing wild type MSH2, L187P and a vector control, were grown to confluency in four 15-cm dishes per cell line. Cells were washed in PBS, and subsequently scraped off the dish in 3 mL of PBS. The cells were harvested by centrifugation and lysed in 1 mL of buffer B for 20 minutes on ice. The lysates were cleared by centrifugation (13000 g, 30 min.), and the supernatants transferred to tubes containing 20 μL TALON metal affinity resin (Clontech). Lysates were incubated for 2 hours at 4°C, after which the beads were washed 4 times in buffer B, followed by addition of 25 μL SDS sample buffer. Samples were prepared in quadruplicates. 17 μL of each sample elution was fractionated by SDS-PAGE (Novex NuPAGE Bis-Tris/MOPS 10% acrylamide) before staining with Coomassie blue, with an estimated protein yield of ~20 μg per lane. Each lane was excised as a single ‘slice’, and tryptic peptides extracted by in gel digestion (0.5μg per slice). Peptide samples were analyzed by LC-MS/MS on a Q Exactive mass spectrometer (Thermo Scientific) coupled to an EASY-nLC 1000 liquid chromatography system (Thermo Scientific) via an EASY-Spray ion source (Thermo Scientific). Peptides were fractionated on a 75 μm x 500 mm EASY-Spray column (Thermo Scientific) over 150 minutes. Precursor ion full scan spectra were acquired over (m/z 300 to 1,800) with a resolution of 70,000 at m/z 400 (target value of 1,000,000 ions, maximum injection time 20 ms). Up to ten data dependent MS2 spectra were acquired with a resolution of 17,500 at m/z 400 (target value of 500,000 ions, maximum injection time 60 ms). Ions with unassigned charge state, and singly or highly (>8) charged ions were rejected. Intensity threshold was set to 2.1 x 10^4^ units. Peptide match was set to preferred, and dynamic exclusion option was enabled (exclusion duration 40 s). Raw MS data files were processed using MaxQuant software (version 1.5.2.8) and searched against UniProtKB human proteome (canonical and isoform sequences). Carbamidomethyl (C) was set as fixed modification and variable modification of acetyl (protein N-term), and oxidized (M) were selected. MaxQuant enzyme specificity was set to trypsin. Lysine and arginine were selected as special amino acid and a maximum number of three missed cleavages were allowed. A minimum peptide length was set to seven residues and a maximum peptide mass was 5,000 Da. A false discovery rate of 1% was set as a threshold at both protein and peptide level, and a mass deviation of 6 parts per million was set for main search and 0.5 Da for MS2 peaks. The match between runs option was selected using the matching and alignment time windows of 0.7 and 20 minutes respectively. Raw intensity values were manually normalized by median ratio of the proteins detected in all samples, and only proteins with four intensities reported in a single set of quadruplicates was carried forward. Zero intensity values were replaced in Persues v 1.5.1.6 (www.biochem.mpg.de/5111810/perseus) from a normal distribution of data based on the input intensities with width and downshift parameters set to default (0.3 and 1.8). Significantly enriched proteins (interactors) were identified by two samples t-test with permutation-based FDR = 0.05 and S0 = 0.2. These are shown as red (103 proteins) among the background of 1483 proteins not satisfying these criteria (see Supporting information S5B Fig).

## Supporting information

S1 Fig MSH2 variants are unstable and not targets of HDAC6. (A) The steady-state level of the indicated wild type (WT) or MSH2 mutants expressed as CFP fusion proteins in U2OS cells were determined using SDS-PAGE and Western blotting with antibodies to GFP (detecting the CFP-tag on MSH2) in cultures that were either treated (+) or untreated (-) with bortezomib for 10 hours. α-tubulin served as a loading control. (B) The steady-state level of the indicated wild type (WT) or MSH2 variants from the OMIM database expressed in U2OS cells was determined using SDS-PAGE and Western blotting with antibodies to 6His-tag on MSH2 in cultures that were either untreated or treated with the proteasome inhibitor bortezomib (BZ) for 10 hours. β-actin served as a loading control. (C) The steady-state level of the indicated wild type (WT) or MSH2 mutants expressed in U2OS cells were determined using SDS-PAGE and Western blotting with antibodies to 6His-tag on MSH2 in cultures that were transfected with either control siRNA (-) or siRNA specific for HDAC6 (+) for 48 hours. α-tubulin served as a loading control. Knock-down efficiency was determined by blotting for HDAC6.
(TIF)

S2 Fig MSH2 quality control is independent of DNA damage and expression level. (A) The amount of wild type MSH2 in U2OS cells was followed in cultures treated with the translation inhibitor cycloheximide (CHX) or the alkylating agent MNNG for 0 or 24 hours. α-tubulin served as a loading control. (B) The degradation of overexpressed wild type MSH2 (left panel) or endogenous MSH2 (right panel) was followed in U2OS cells at 37°C after treating with cycloheximide (CHX) for 0, 8, 16 and 24 hours. α-tubulin served as a loading control. The half-life (t½) was determined by densitometry and is given below along with the standard deviation (C) The expression level of stably transfected MSH2 variants was determined by blotting using antibodies to MSH2 and to the 6His-tag on the recombinant MSH2 variants. β-actin served as a loading control. Note that the recombinant MSH2 variants are not overexpressed compared to the endogenous MSH2 protein. (D) The degradation of the MSH2 variants stably transfected in U2OS cells was followed after treating with cycloheximide (CHX) for 0, 8, 16 and 24 hours. β-actin served as a loading control.
(TIF)

S3 Fig The ubiquitin-proteasome system appears normal at 29°C. The cellular levels of ubiquitin-protein conjugates and 26S proteasomes was compared between U2OS cells grown at 29°C and at 37°C by blotting for ubiquitin (Ub), the 19S regulatory complex subunit Rpn1 and the 20S proteasome α-subunits. α-tubulin served as a loading control.
(TIF)

S4 Fig The MSH2 subcellular localization is not affected by the introduced missense mutations. (A) The subcellular localization of wild type MSH2 (left panels) and, as an example of a MSH2 mutant, C199R (right panels) were determined using antibodies to the 6His-tagged MSH2. Hoechst staining was used to mark the nucleus. (B) Quantification of nuclear MSH2 localization determined from stains as shown in (a) for wild type (WT) MSH2 and the selected MSH2 mutants. Between 100 and 1000 cells were included for each quantification. The error bars indicate the standard deviation.
(TIF)

S5 Fig Mass spectrometry analysis of the wild type and mutant MSH2 interactomes. (A) U2OS cells expressing vector, 6His-tagged wild type MSH2 or 6His-tagged MSH2-L187P were used for precipitation experiments with metal affinity beads in quadruplicates (only one of four experiments is shown). The precipitated material was separated by SDS-PAGE and the gel stained with Coomassie Brilliant Blue (CBB). (B) Scatter plot showing the 1586 proteins that were identified and quantified in the three sets of quadruplicates (as described in materials and methods). Axes are log2 values of the protein intensity ratio with control purifications of wild type MSH2 purifications (x-axis) and L187P MSH2 purification (y-axis). Points are calculated from the ratio of the arithmetic mean of the four intensities from each quadruplicate group. Density of data in the chart is represented by color from cyan (most dense) to green (least). Line representing y = x (broken red), and MSH2, MSH3 and MSH6 proteins are indicated. (C) Column chart showing average log2 wild type/L187P ratios for all proteins together (‘All proteins’, n = 1586), or for the individual MSH proteins (as indicated). Each replicate was used to produce a single ratio by comparison with the other group, providing four ratios. Error bars represent standard deviation of these ratios for MSH2, MSH3 and MSH6 proteins, or the average standard deviation of all proteins (‘All proteins’). p-value of the two samples t-test comparing difference between wt and L187P MSH2 purifications is shown above each column. (D) Metal affinity co-precipitation of 6His-MSH2 and 6His-MSH2-L187P. The precipitated material was analyzed by SDS-PAGE and Western blotting using antibodies to the 6His-tag (on MSH2), MSH3 and MSH6. Input samples (5%) were included as a control. α-tubulin served as a loading control.
(TIF)

S6 Fig Comparison of FoldX calculations with other disease predictors. (A) Comparison of the predicted pathogenicity of MSH2 variants by ΔΔG and PolyPhen2 (left), and ΔΔG and PROVEAN (right). The variants are colored by dataset: short half-life (t½, red), intermediate (yellow), and long t½ (light green) for variants tested in this work; common variants as observed in ExAC (Lek 2016) (dark green, see also Fig 6C in the main manuscript), known non-pathogenic (blue) and pathogenic (purple) MSH2 variants from the literature, and recently identified pathogenic variants (RIPV, magenta) according to Houlleberghs *et al*., 2016. (B) Score distributions for the sequence-based predictors we tested. For SIFT and PROVEAN, the default thresholds for pathogenic mutations (<log(0.05) for SIFT, <-2.5 for PROVEAN) are indicated with a horizontal line. Note that low scores in the SIFT plot indicate putative pathogenic mutations, while high scores in the PolyPhen2, PROVEAN, Rosetta, CADD, and co-variation plots are considered pathogenic. All predictors give correct trends, with low half-life variants scoring worse on average than intermediate and long half-life MSH2 variants. However, neither predictor separates these groups as well as FoldX ΔΔGs (Fig 6A). (C) Receiver operating characteristic (ROC) curves for all methods used in this work, plus a simple linear model of predicting stability changes from a multiple sequence alignment (MSA) of MSH2 (green). (D) Leave-one-out cross validation was performed to assess the stability and predictive power of pathogenicity prediction. The plot shows accuracy of prediction of the left-out mutation across the leave-one-out procedure for each method. As in the ROC curve in Fig 6B, co-variation and the biophysical models Rosetta and FoldX perform better than the more established sequence-based predictors PROVEAN, SIFT, CADD, and PolyPhen2.
(TIF)

S1 File Saturation mutagenesis dataset as spreadsheet. (XLSX)

S2 File Saturation mutagenesis dataset as heatmap. (PDF)

S3 File Comparison of predictions for the selected MSH2 variants. (XLSX)

S4 File Mass spectrometry dataset. (XLSX)
