# Supplementary figures and images for: Predicting the impact of Lynch syndrome-causing missense mutations from structural calculations
Source: PLoS Genet. 2017 Apr 19;13(4):e1006739. doi: 10.1371/journal.pgen.1006739 (PMC5415204; doi:10.1371/journal.pgen.1006739)

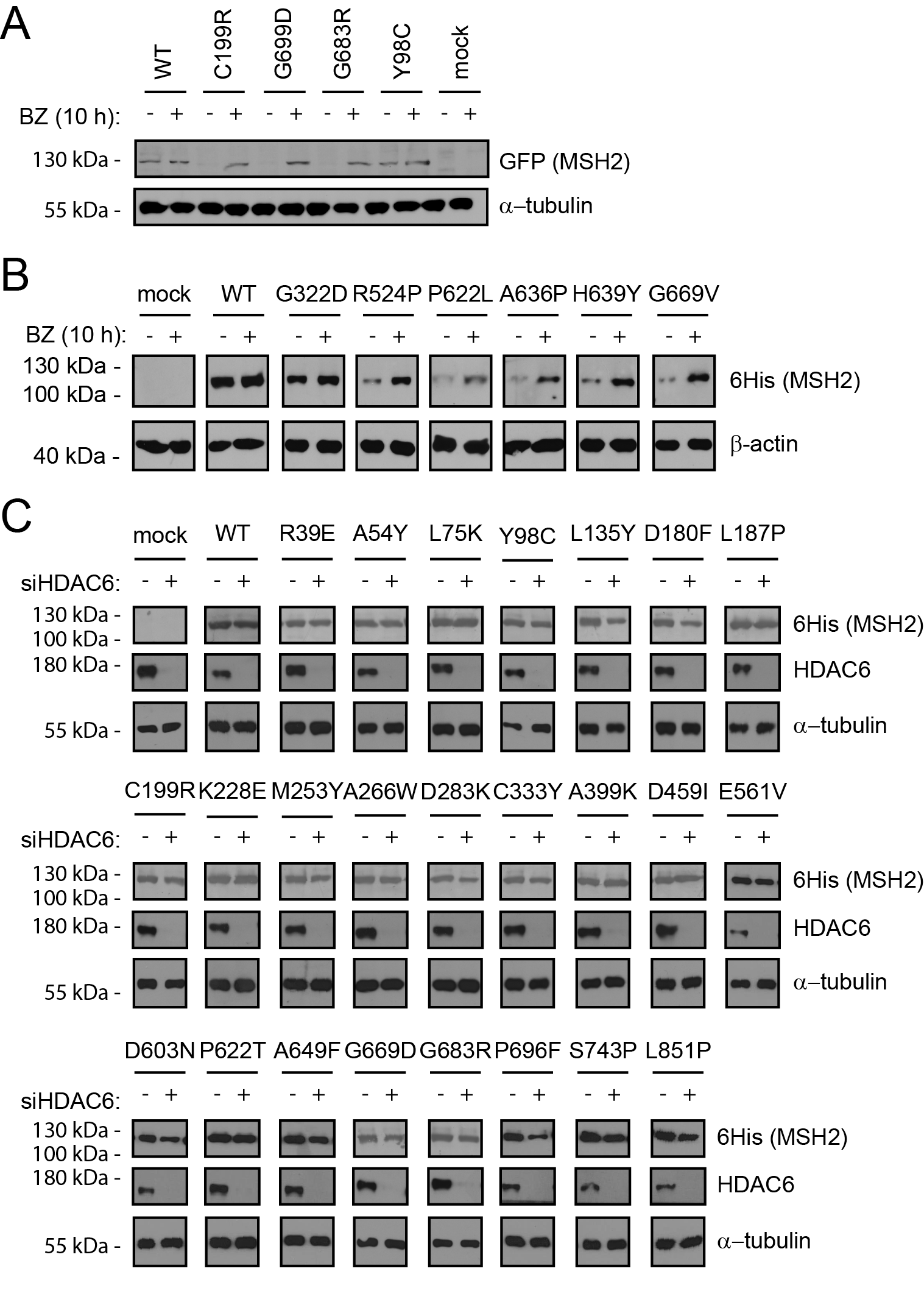

Supplement: S1 Fig — (A) The steady-state level of the indicated wild type (WT) or MSH2 mutants expressed as CFP fusion proteins in U2OS cells were determined using SDS-PAGE and Western blotting with antibodies to GFP (detecting the CFP-tag on MSH2) in cultures that were either treated (+) or untreated (-) with bortezomib for 10 hours. α-tubulin served as a loading control. (B) The steady-state level of the indicated wild type (WT) or MSH2 variants from the OMIM database expressed in U2OS cells was determined using SDS-PAGE and Western blotting with antibodies to 6His-tag on MSH2 in cultures that were either untreated or treated with the proteasome inhibitor bortezomib (BZ) for 10 hours. β-actin served as a loading control. (C) The steady-state level of the indicated wild type (WT) or MSH2 mutants expressed in U2OS cells were determined using SDS-PAGE and Western blotting with antibodies to 6His-tag on MSH2 in cultures that were transfected with either control siRNA (-) or siRNA specific for HDAC6 (+) for 48 hours. α-tubulin served as a loading control. Knock-down efficiency was determined by blotting for HDAC6. (TIF) [file pgen.1006739.s001.tif]

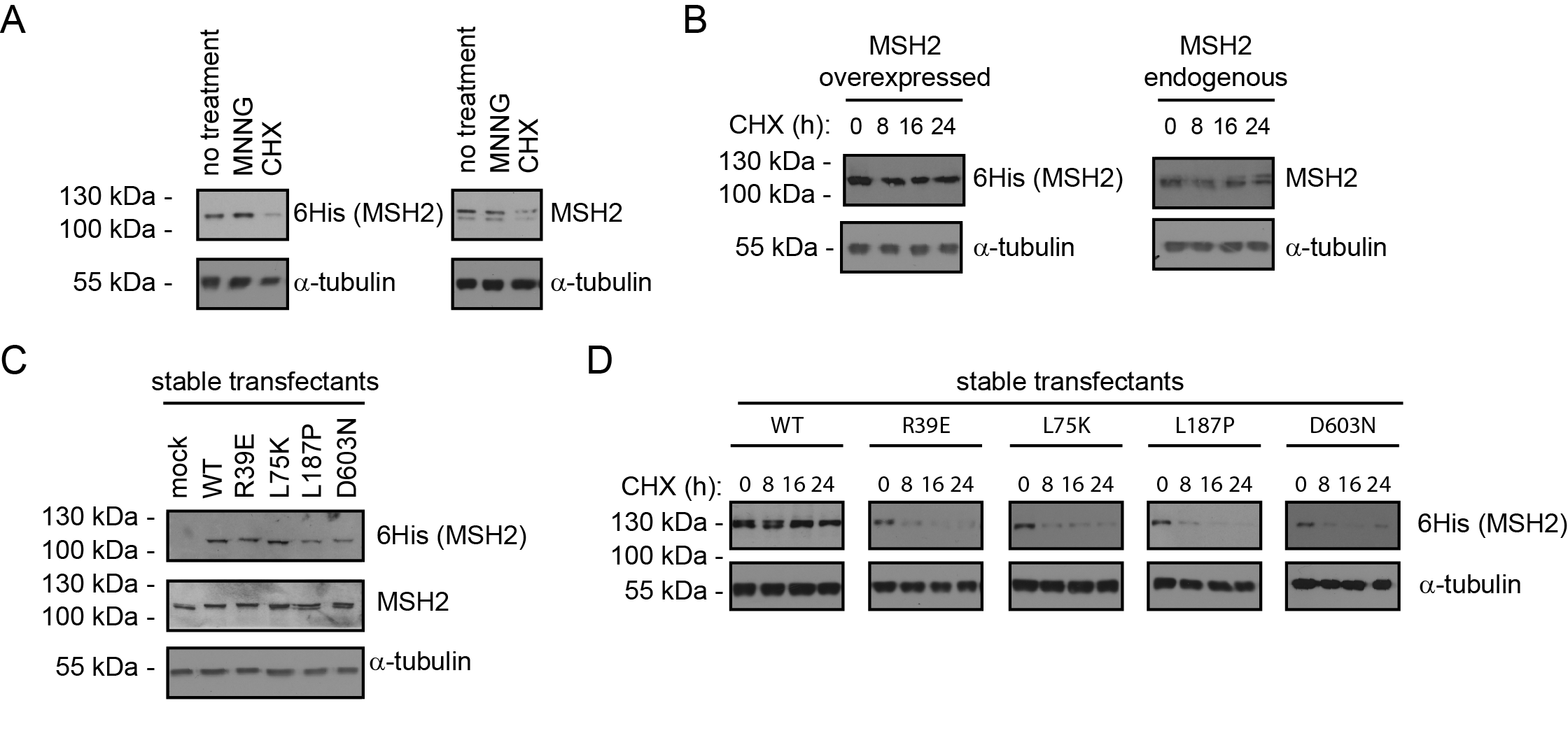

Supplement: S2 Fig — (A) The amount of wild type MSH2 in U2OS cells was followed in cultures treated with the translation inhibitor cycloheximide (CHX) or the alkylating agent MNNG for 0 or 24 hours. α-tubulin served as a loading control. (B) The degradation of overexpressed wild type MSH2 (left panel) or endogenous MSH2 (right panel) was followed in U2OS cells at 37°C after treating with cycloheximide (CHX) for 0, 8, 16 and 24 hours. α-tubulin served as a loading control. The half-life (t½) was determined by densitometry and is given below along with the standard deviation (C) The expression level of stably transfected MSH2 variants was determined by blotting using antibodies to MSH2 and to the 6His-tag on the recombinant MSH2 variants. β-actin served as a loading control. Note that the recombinant MSH2 variants are not overexpressed compared to the endogenous MSH2 protein. (D) The degradation of the MSH2 variants stably transfected in U2OS cells was followed after treating with cycloheximide (CHX) for 0, 8, 16 and 24 hours. β-actin served as a loading control. (TIF) [file pgen.1006739.s002.tif]

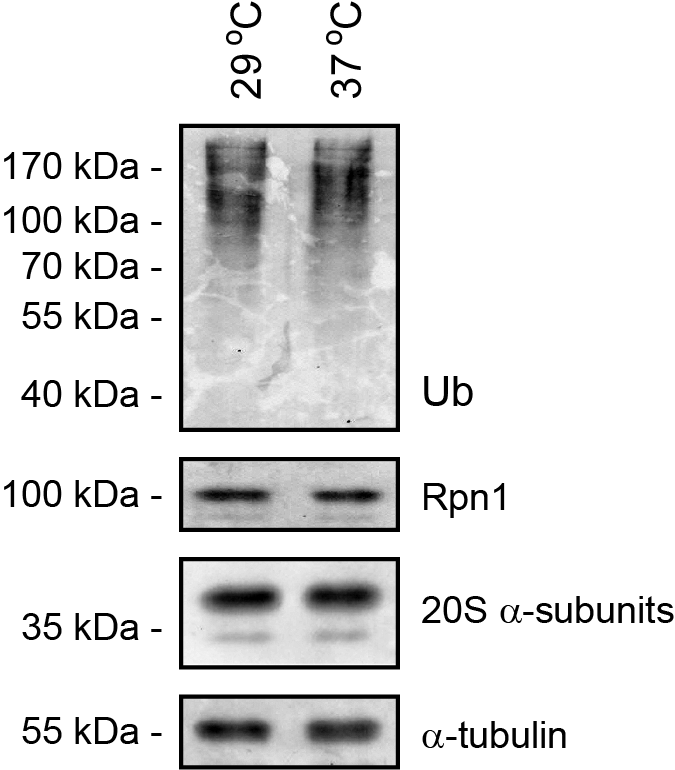

Supplement: S3 Fig — The cellular levels of ubiquitin-protein conjugates and 26S proteasomes was compared between U2OS cells grown at 29°C and at 37°C by blotting for ubiquitin (Ub), the 19S regulatory complex subunit Rpn1 and the 20S proteasome α-subunits. α-tubulin served as a loading control. (TIF) [file pgen.1006739.s003.tif]

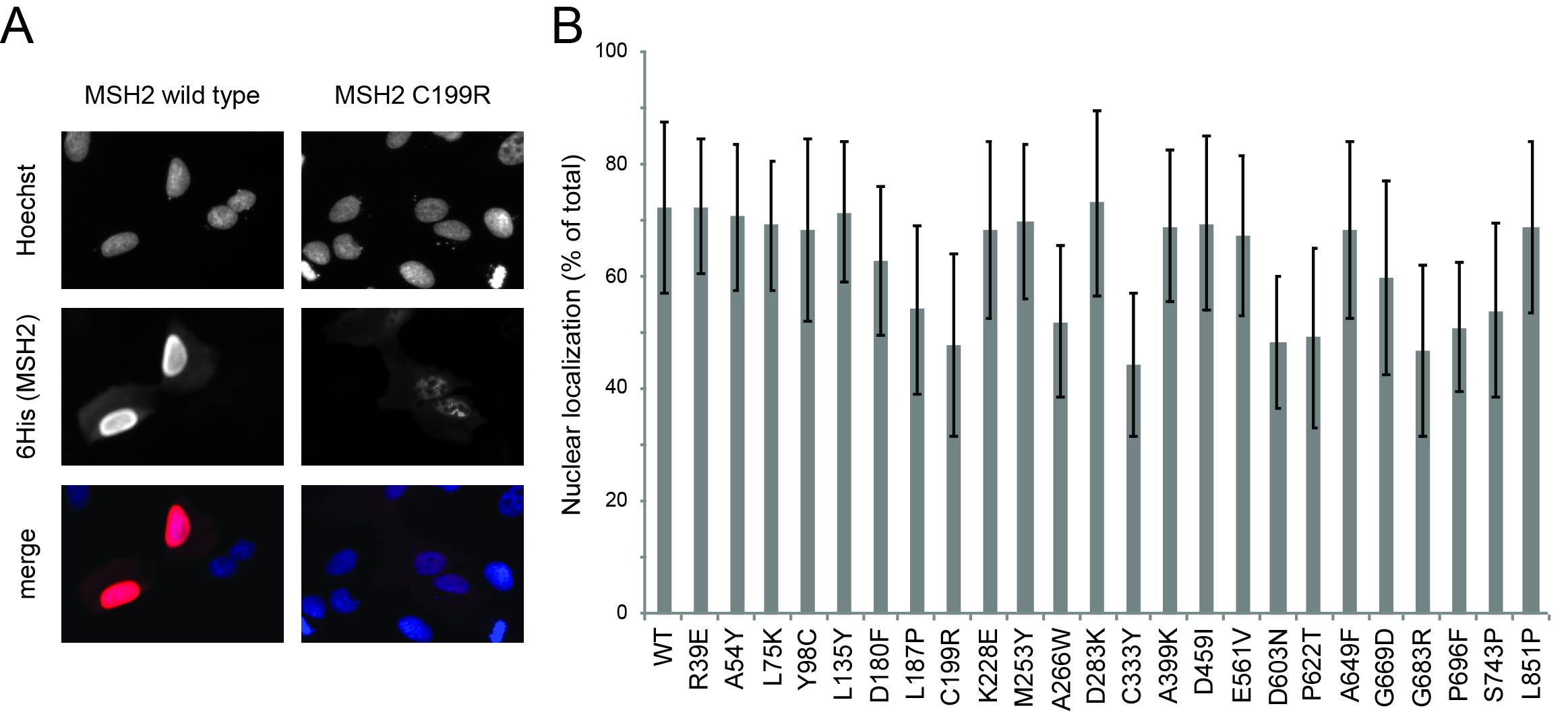

Supplement: S4 Fig — (A) The subcellular localization of wild type MSH2 (left panels) and, as an example of a MSH2 mutant, C199R (right panels) were determined using antibodies to the 6His-tagged MSH2. Hoechst staining was used to mark the nucleus. (B) Quantification of nuclear MSH2 localization determined from stains as shown in (a) for wild type (WT) MSH2 and the selected MSH2 mutants. Between 100 and 1000 cells were included for each quantification. The error bars indicate the standard deviation. (TIF) [file pgen.1006739.s004.tif]

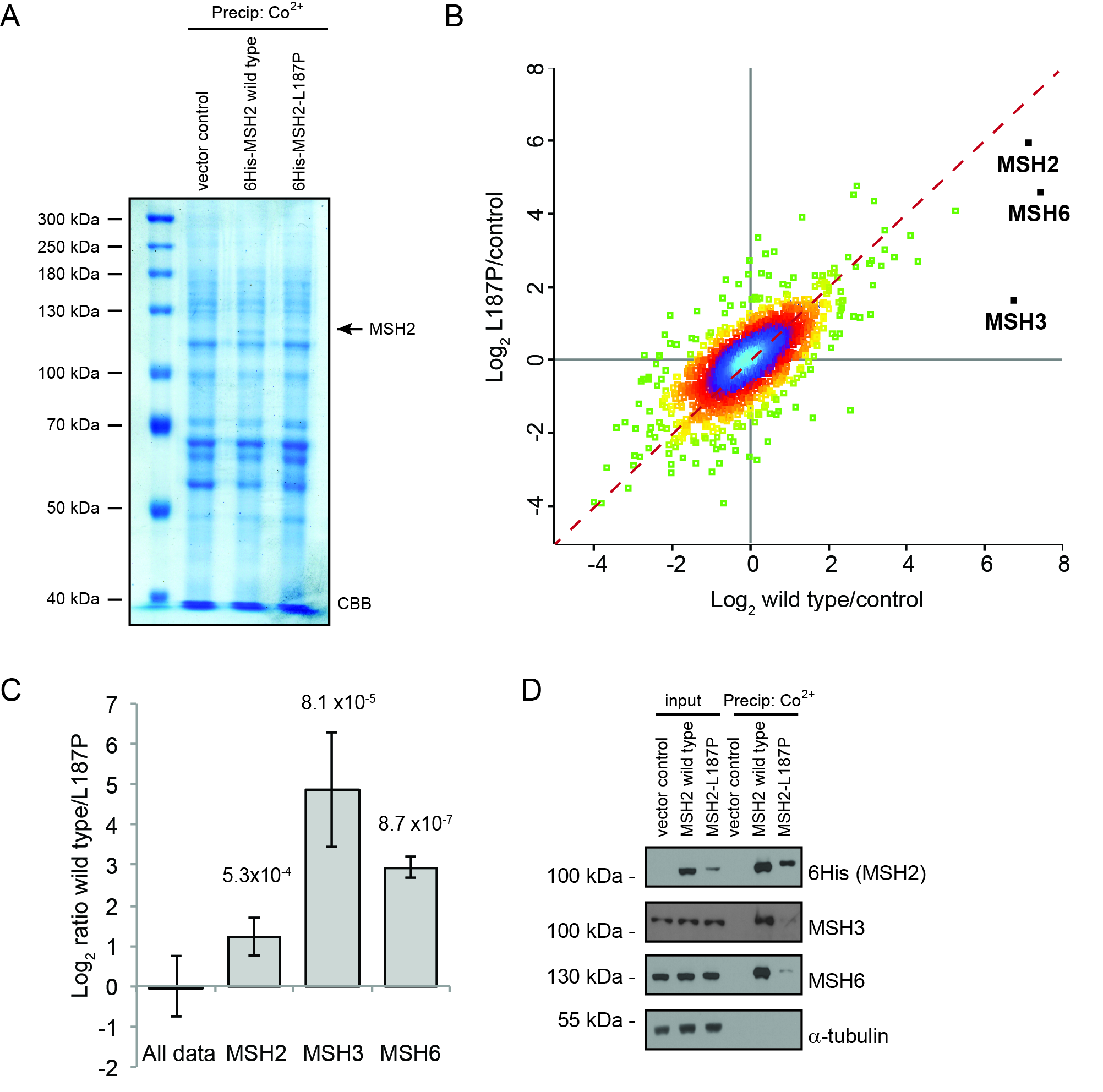

Supplement: S5 Fig — (A) U2OS cells expressing vector, 6His-tagged wild type MSH2 or 6His-tagged MSH2-L187P were used for precipitation experiments with metal affinity beads in quadruplicates (only one of four experiments is shown). The precipitated material was separated by SDS-PAGE and the gel stained with Coomassie Brilliant Blue (CBB). (B) Scatter plot showing the 1586 proteins that were identified and quantified in the three sets of quadruplicates (as described in materials and methods). Axes are log2 values of the protein intensity ratio with control purifications of wild type MSH2 purifications (x-axis) and L187P MSH2 purification (y-axis). Points are calculated from the ratio of the arithmetic mean of the four intensities from each quadruplicate group. Density of data in the chart is represented by color from cyan (most dense) to green (least). Line representing y = x (broken red), and MSH2, MSH3 and MSH6 proteins are indicated. (C) Column chart showing average log2 wild type/L187P ratios for all proteins together (‘All proteins’, n = 1586), or for the individual MSH proteins (as indicated). Each replicate was used to produce a single ratio by comparison with the other group, providing four ratios. Error bars represent standard deviation of these ratios for MSH2, MSH3 and MSH6 proteins, or the average standard deviation of all proteins (‘All proteins’). p-value of the two samples t-test comparing difference between wt and L187P MSH2 purifications is shown above each column. (D) Metal affinity co-precipitation of 6His-MSH2 and 6His-MSH2-L187P. The precipitated material was analyzed by SDS-PAGE and Western blotting using antibodies to the 6His-tag (on MSH2), MSH3 and MSH6. Input samples (5%) were included as a control. α-tubulin served as a loading control. (TIF) [file pgen.1006739.s005.tif]

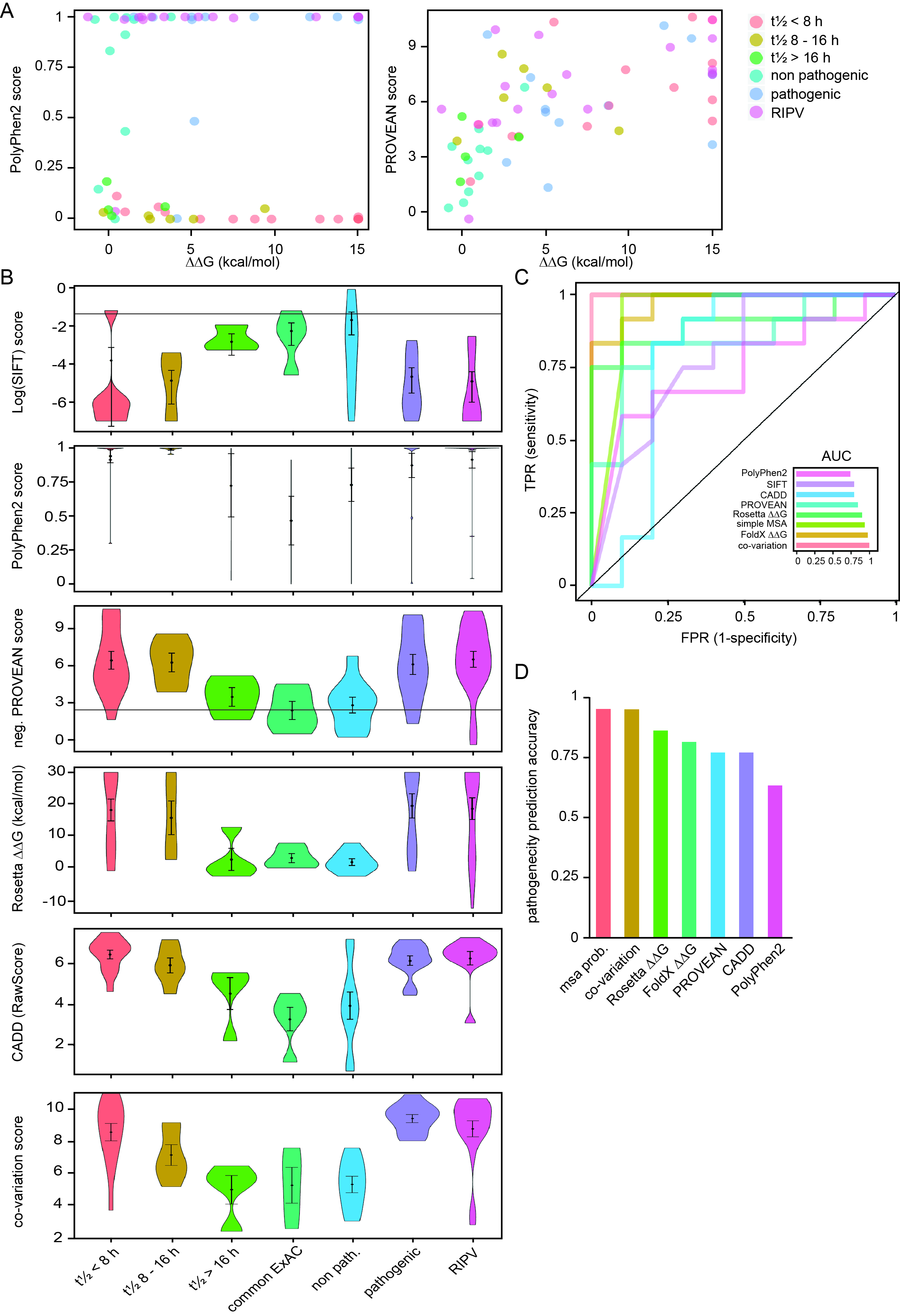

Supplement: S6 Fig — (A) Comparison of the predicted pathogenicity of MSH2 variants by ΔΔG and PolyPhen2 (left), and ΔΔG and PROVEAN (right). The variants are colored by dataset: short half-life (t½, red), intermediate (yellow), and long t½ (light green) for variants tested in this work; common variants as observed in ExAC (Lek 2016) (dark green, see also Fig 6C in the main manuscript), known non-pathogenic (blue) and pathogenic (purple) MSH2 variants from the literature, and recently identified pathogenic variants (RIPV, magenta) according to Houlleberghs et al., 2016. (B) Score distributions for the sequence-based predictors we tested. For SIFT and PROVEAN, the default thresholds for pathogenic mutations (<log(0.05) for SIFT, <-2.5 for PROVEAN) are indicated with a horizontal line. Note that low scores in the SIFT plot indicate putative pathogenic mutations, while high scores in the PolyPhen2, PROVEAN, Rosetta, CADD, and co-variation plots are considered pathogenic. All predictors give correct trends, with low half-life variants scoring worse on average than intermediate and long half-life MSH2 variants. However, neither predictor separates these groups as well as FoldX ΔΔGs (Fig 6A). (C) Receiver operating characteristic (ROC) curves for all methods used in this work, plus a simple linear model of predicting stability changes from a multiple sequence alignment (MSA) of MSH2 (green). (D) Leave-one-out cross validation was performed to assess the stability and predictive power of pathogenicity prediction. The plot shows accuracy of prediction of the left-out mutation across the leave-one-out procedure for each method. As in the ROC curve in Fig 6B, co-variation and the biophysical models Rosetta and FoldX perform better than the more established sequence-based predictors PROVEAN, SIFT, CADD, and PolyPhen2. (TIF) [file pgen.1006739.s006.tif]

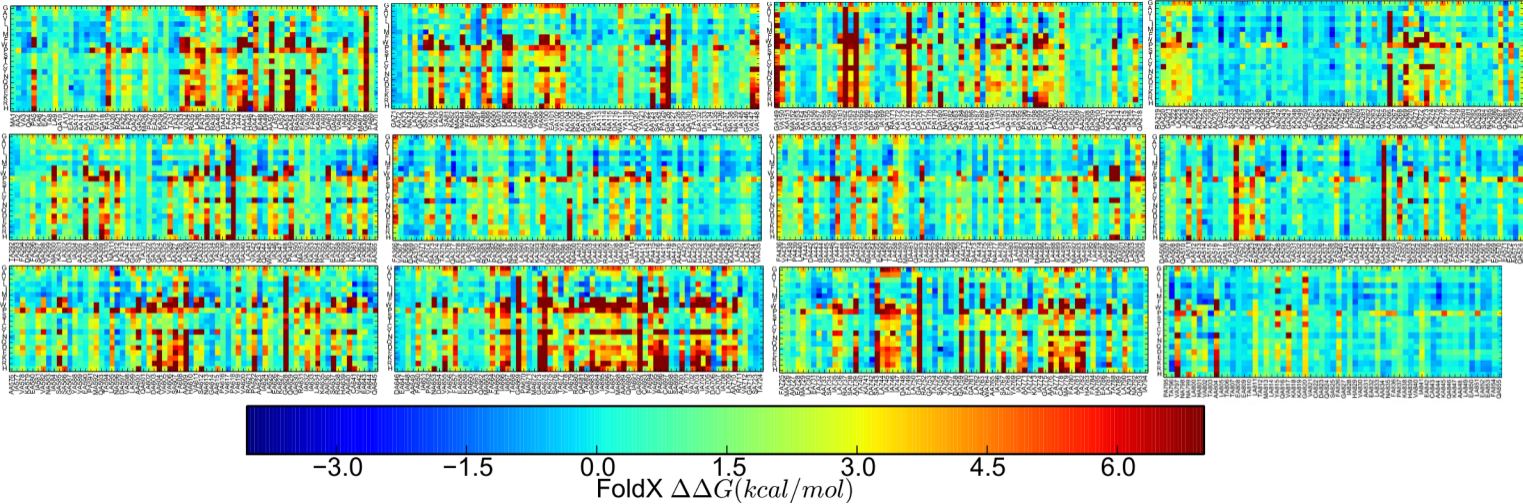

Supplement: S2 File — (PDF) [file pgen.1006739.s008.pdf]
